# Bioactivity of Size-Fractionated and Unfractionated Humic Substances From Two Forest Soils and Comparative Effects on N and S Metabolism, Nutrition, and Root Anatomy of *Allium sativum* L

**DOI:** 10.3389/fpls.2020.01203

**Published:** 2020-08-14

**Authors:** Diego Pizzeghello, Michela Schiavon, Ornella Francioso, Francesca Dalla Vecchia, Andrea Ertani, Serenella Nardi

**Affiliations:** ^1^ Dipartimento di Agronomia, Animali, Alimenti, Risorse naturali e Ambiente, Università degli Studi di Padova, Legnaro, Italy; ^2^ Dipartimento di Scienze e Tecnologie Agro-Alimentari, Università di Bologna, Bologna, Italy; ^3^ Dipartimento di Biologia, Università degli Studi di Padova, Padova, Italy; ^4^ Dipartimento di Scienze Agrarie, Forestali e Alimentari, Università di Torino, Torino, Italy

**Keywords:** humic substances, molecular size, biostimulants, growth, sulfur, nitrogen, amino acids, root cell differentiation

## Abstract

Humic substances (HS) are powerful natural plant biostimulants. However, there is still a lack of knowledge about the relationship between their structure and bioactivity in plants. We extracted HS (THE1-2) from two forest soils covered with *Pinus mugo* (1) or *Pinus sylvestris* (2). The extracts were subjected to weak acid treatment to produce size-fractionated HS (high molecular size, HMS1-2; low molecular size, LMS1-2). HS were characterized for total acidity, functional groups, element and auxin (IAA) contents, and hormone-like activity. HS concentrations ranging from 0 to 5 mg C L^−1^ were applied to garlic (*Allium sativum* L.) plantlets in hydroponics to ascertain differences between unfractionated and size-fractionated HS in the capacity to promote mineral nutrition, root growth and cell differentiation, activity of enzymes related to plant development (invertase, peroxidase, and esterase), and N (nitrate reductase, glutamine synthetase) and S (O-acetylserine sulphydrylase) assimilation into amino acids. A positive linear dose-response relationship was determined for all HS in the range 0–1 mg C L^−1^, while higher HS doses were less effective or ineffective in promoting physiological-biochemical attributes of garlic. Bioactivity was higher for size-fractionated HS according to the trend LMS1-2>HMS1-2>THE1-2, with LMS2 and HMS2 being overall more bioactive than LMS1 and HMS1, respectively. LMS1-2 contained more N, oxygenated functional groups and IAA compared to THE1-2 and HMS1-2. Also, they exhibited higher hormone-like activities. Such chemical properties likely accounted for the greater biostimulant action of LMS1-2. Beside plant growth, nutrition and N metabolism, HS stimulated S assimilation by promoting the enrichment of garlic plantlets with the S amino acid alliin, which has recognized beneficial properties in human health. Concluding, this study endorses that i) treating THE with a weak acid produced sized-fractionated HS with higher bioactivity and differing in properties, perhaps because of novel molecular arrangements of HS components that better interacted with garlic roots; ii) LMS from forest soils covered with *P. mugo* or *P. sylvestris* were the most bioactive; iii) the cover vegetation affected HS bioactivity iv); HS stimulated N and S metabolism with relevant benefits to crop nutritional quality.

## Introduction

A great deal of literature has long corroborated a key role of soil organic matter (SOM) in soil preservation and fertility (Nardi et al., 2004; Johnston et al., 2009). However, a number of factors, primarily ongoing climate changes, extensive farming and industrial agriculture, have progressively led to a dramatic decrease of SOM content, thus hampering soil fertility and crop yield goals (Kopittke et al., 2019).

To overcome the issue of soil fertility decline, innovative technologies have been proposed that make use of substances with biostimulant properties (Ertani et al., 2009; du Jardin, 2015; Colla et al., 2016; Nardi et al., 2016; Canellas et al., 2019). According to the definition formulated by Yakhin et al. (2017), biostimulants must be intended as products of biological origin able to afford benefits to plants, by stimulating their productivity and inducing positive physiological responses under sub-optimal nutritional inputs and environmental stresses. The effects of biostimulants in plants are not due to the sole occurrence of endogenous plant growth promoters or stress signaling and protective compounds, but are the result of joined properties of the mixture constituents (Nardi et al., 2016; Yakhin et al., 2017; Rouphael and Colla, 2018). Biostimulants can be grouped in two main categories, microbial and non-microbial (Regulation EU, 2019/1009). The former group includes beneficial fungi and bacteria, the latter humic substances, protein hydrolysates and various N-compounds, seaweed extracts and botanicals, beneficial elements, chitosan and other biopolymers.

Humic substances (HS) in particular, represent pivotal and abundant components of SOM (until 80%), capable of coordinating physical, chemical and biological processes in soil through the control of ion bioavailability (Zanin et al., 2019). Their nature is contentious, but the hypothesis that HS could be artifacts resulting from alkaline extraction procedures has been definitely rejected (Nebbioso and Piccolo, 2012). Early theories asserted that HS possess a macromolecular structure producing random coil conformations (Swift, 1999) or micelles or “pseudo micellar” structures (Wershaw, 1999) in solution. Later, other authors argued that HS are a buildup of small heterogenous organic molecules, such as sugars, fatty acids, polypeptides, aliphatic chains, and aromatic rings, hold together and stabilized by intermolecular hydrophobic interactions and hydrogen bonds (Piccolo, 2001; Piccolo, 2002; Sutton and Sposito, 2005; Šmejkalová and Piccolo, 2008).

Some constituents of HS and their arrangements can dictate the establishment of biotic rhizosphere associations and provoke plant responses *via* interaction with receptors localized at the root cell membranes (Shah et al., 2018). HS act as biostimulants owing their capacity to prompt plant growth and provide benefits to the primary metabolism and biochemical pathways involved in the synthesis of secondary compounds, primarily phenolics (Nardi et al., 2017; Nardi et al., 2018; Nunes et al., 2019; Zanin et al., 2019). The effects of HS on plant growth are mainly due to their capacity to increase soil micro- and macro- element availability for plant uptake by forming soluble complexes with several ions (Garcia-Mina et al., 2004; Varanini and Pinton, 2006). Furthermore, HS can induce changes in the perception of the plant nutrient status and in the signaling pathways implied in nutrient sensing, thus speeding up nutrient transport processes in the roots through increased synthesis and induced activity of plasma membrane localized-nutrient transporters and H^+^-ATPases (Nardi et al., 1991; Chen et al., 2004; Zandonadi et al., 2007; Canellas and Olivares, 2014; Zanin et al., 2018; Canellas et al., 2019; Olaetxea et al., 2019). The activation of H^+^-ATPase by HS seems to depend on mechanisms that use nitric oxide (NO) as a messenger during the early stages of lateral root emergence (Zandonadi et al., 2010).

Some authors ascribe part of HS bioactivity to the content in hormones and/or to the hormone-like activity displayed by certain HS functional constituents, such as aliphatic-C, carboxyl-C, and phenol-C (aromatic) groups (Schiavon et al., 2010; Canellas et al., 2011; Pizzeghello et al., 2013; Nardi et al., 2018). The HS biological activity was also ascertained to rely on HS hydrophobic features (Canellas et al., 2009; Martínez Balmori et al., 2014) and molecular distribution (Piccolo et al., 1992; Muscolo et al., 2007; Nardi et al., 2007; Zandonadi et al., 2007; Canellas et al., 2010).

The linkage between HS stimulation effects in plants and HS molecular dimension is however under debate, as contrasting results have been produced so far. Piccolo (2001), based on early evidence (Dell’Agnola and Nardi, 1987; Nardi et al., 1988; Nardi et al., 1991), stated that HS properties result from HS treatment with organic acids. Such assumption was explained in terms of permanent alteration of the hydrophobic domains in the micelle-like aggregations, with the shift from high molecular size (HMS) to small molecular size (LMS) due to disaggregation effects caused by organic acids (Piccolo et al., 1996a; Piccolo et al., 1996b). Under this condition, the carboxyl groups of organic acids are oriented between the micelles and the water interface.

In many studies, the LMS fraction of HS is reported to be the most effective in inducing stimulatory responses in plants (Nardi et al., 1988; Piccolo et al., 1992; Muscolo et al., 2007; Nardi et al., 2007). Nevertheless, the HMS fraction proved to behave as a very positive root growth regulator in other works (Zandonadi et al., 2007; Dobbss et al., 2007; Canellas et al., 2009; Schiavon et al., 2010). The hypothesized mechanisms through which the two fractions induce plant physiological responses sturdily differ. While the LMS fraction might be able to enter the root cells and elicit intracellular molecular signals (Muscolo et al., 2007), the HMS fraction is believed to trigger hormone signaling pathways inside cells after binding to root cell membrane external protein receptors (Dobbss et al., 2007; Muscolo et al., 2007; Schiavon et al., 2010).

The HMS/LMS ratio varies based on soil type. In forest soils, the biological activity persists at minimum and a thick humus layer is formed as a result of SOM accumulation. More than 90% of this SOM consists of humus derived from intense decomposition of plant and animal litters, with rates varying for the different litter types according to the substrate quality. Fine roots and deciduous leaves that are high in nutrients and possess fungal and bacterial necromass can be decomposed in one year, while most coniferous leaves (needles) require some years to decay, and decades (branches) to centuries (tree trunks) to disappear, depending on pedoclimatic conditions (Breemen and Buurman, 2002). The small molecules derived from organic matter degradation become self-reassembled in molecular clouds, with properties that reflect those of their parental materials (Ponge, 2015). Studies conducted on bulk forest soil samples and HS fractions indicated that different forest humus types result from different rates, but common pathways, of litter decomposition (Ziegler et al., 1992). This humus is recognized as a rich source of HS that may be used as valuable biostimulants in agriculture.

To date, differences in biological activity of HS derived from forest soils have not been largely studied (Nardi et al., 2000; Pizzeghello et al., 2001; Pizzeghello et al., 2002). Also, despite all relevant findings on HS reported so far, the nexus between of HS bioactivity and chemical structure still represents a complex, controversial and partially unknown phenomenon that deserves more investigation (Calderín García et al., 2016; Calderín García et al., 2019). Therefore, this study is aimed at: i) assaying differences in chemical composition and biological activity between unfractionated and fractionated HS extracted from two forest soils subjected to different vegetation cover; ii) appraising differential effects of these HS in altering root nutrient content status, primary metabolism and ultrastructure of garlic (*Allium sativum* L.) over a short-time; iii) determining a dose-response relationship for both unfractionated and fractionated HS.

Garlic was used in this study because is a common and relevant horticultural crop worldwide, rich in healthy phytochemicals, including certain sulfur (S)-compounds. To date, the effect of HS on S metabolism has been poorly investigated and we aimed to assay whether short-term HS application to garlic plantlets could positively impact on it.

## Materials and Methods

### Extraction and Fractionation of Humic Substances

Humic substances (HS) appraised in this study were extracted from two forest soils (Rendzic Leptosols) (IUSS WRB, 2015) derived from our Pedoteca and originally collected at Cortina d’Ampezzo (NE Italy 46°83’ N, 12°80’ E). The two soils were designed with (1) and (2) depending on the main vegetation cover, consisting of *Pinus mugo* and *Pinus sylvestris*, respectively. HS extraction and purification procedures were performed as previously described by Nardi et al. (2000). Briefly, HS were extracted in 0.1 M KOH (1:20 w/v) at room temperature for 16 h under a N_2_ atmosphere. Alkaline extracts were dialyzed against double-distilled water using a dialysis membrane tubing with a molecular weight cut-off (MWCO) of 18,000 Da (Visking, London), and then completely desalted through an Amberlite IR 120 [H^+^] cation exchange resin (Merck, Milan, Italy).

To fractionate HS, aliquots of each total humic extract (THE) (>18,000 MWCO) were treated with glacial acetic acid (99%) (Merck) until pH 2.1 was achieved, and further dialyzed against deionized distilled water by MWCO 3,500 Spectrapore 3 tubing (Spectrum, Gardena, CA). Two different nominal molecular size fractions, high (>3,500, HMS) and low (<3,500, LMS), were obtained (Nardi et al., 2000) from each THE. The two total humic extracts and their relative high and low size fractions were named as THE1, HMS1, LMS1 when deriving from the soil covered by *P. mugo*, and as THE2, HMS2, LMS2 when obtained from the soil covered by *P. sylvestris*.

### Chemical, Spectroscopic and Biological Characterization of Humic Substances

The elemental composition (C, H, N, and S) of total humic extracts (THE) and their fractions (HMS and LMS) was determined using an elemental analyzer (Thermo Electron model EA 1110 Waltham, MA, USA), while oxygen content was computed by subtraction. The total acidity and the content of carboxylic groups were determined according to the procedure proposed by Swift (1996), while the content of phenol-OH groups was calculated by difference. The 13C NMR (Nuclear Magnetic Resonance) spectra were recorded by a Bruker AMX-500 spectrometer (Bruker, Kaarlsruhe, Germany), using inverse-gated decoupling experiments for quantitative intensity distribution. Further details regarding this analysis are reported by Nardi et al. (2000). The degree of aromaticity (AD), and HB (hydrophobic C content):HI (hydrophilic C content) ratio (hydrophobicity index) were used as variables to describe the HS in the multivariate analysis. AD was calculated using the formula: (105–165)/(105–0). HI/HB was computed based on the formula: [(48–105) + (165–190)]/[(0–48) + (105–165)]. The areas of the 0–48 and 105–165 ppm regions were used to calculate the hydrophobicity (HB) of the HS, whereas those of the 48–105 and 160–190 ppm regions were used to obtain the hydrophilicity (HI) of the HS. The contributions of C functional groups were divided into different chemical-shift areas: 165–190 ppm (carboxyl-C), 145–165 ppm phenolic C, 110–165 ppm (aromatic-C), 48–105 ppm (protein and anomeric C) and 0–48 ppm (alkyl-C) (Nardi et al., 2000). The integration of the peaks within each of the chemical shift regions allowed the evaluation of the relative C contents expressed as percentages of the total area.

The amount of indole-3-acetic acid (IAA) in HS (total humic extracts and relative HMS and LMS fractions) was estimated by enzyme linked immunosorbent assay (ELISA) (Phytodetek-IAA, Merck). HS were then examined for auxin-, gibberellin - and cytokinin-like activity (Audus, 1972; Pizzeghello et al., 2001; Pizzeghello et al., 2013). The IAA-like activity in particular, was estimated by measuring the reduction of watercress (*Lepidium sativum* L.) root length after treatment with either IAA or HS. Conversely, the gibberellin-like (GA-like) activity was determined by evaluating increases in length of lettuce (*Lactuca sativa* L.) epicotyls following application of GA and HS (Audus, 1972). Specifically, watercress and lettuce seeds were surface-sterilized by soaking in 8% (v/v) hydrogen peroxide for 15 min. After rinsing five times with sterile distilled water, seeds were placed on sterile filter papers inside sterile Petri dishes (10 seeds per dish). For watercress, the filter paper was wetted with 1.2 mL of 1 mM CaSO_4_ (control), or 1.2 mL of 0.1, 1, 10, 20 mg L^−1^ IAA solution (Merck, Milan, Italy) for the calibration curve, or 1.2 mL of a serial dilution of HS. For lettuce, the experimental design was the same as described for watercress except that the sterile filter paper was wetted with 1.4 mL, and the calibration curve was a progression of 0.1, 1, 10, 100 mg L^−1^ GA solution (Merck, Milan, Italy). The seeds were hold inside a germination room in the dark at 25°C. After 48 h for watercress and 72 h for lettuce, seedlings were removed and the root or epicotyl lengths were measured using a TESA-CAL IP67 electronic calibre (TESA, Renens, Switzerland) and Data Direct software, version 1 (ArtWare, Asti, Italy). Data were transformed on natural logarithmic scale to obtain the best linear fitting. The cytokinin-like activity was evaluated by weighing the cotyledons of radish (*Raphanus sativus* L.) seedlings (Pizzeghello et al., 2013). Seeds of *R. sativus* were germinated on wet paper towels hold for 2 to 3 days in the dark at 22 to 26°C. From each seedling, the smaller cotyledon was excised carefully removing all tissues of petiole, and then placed onto a Whatman No. 1 filter paper settled at the bottom of a 9-cm diameter Petri dish (density = 15 cotyledons per petri dish). The filter paper was imbibed with: i) cytokinin isopentenyladenosine (IPA) (20, 40, 60, and 80 µM) dissolved in 95% ethanol or ii) 95% ethanol only (controls), followed by evaporation of the ethanol under an IR lamp, or iii) with HS (0.1, 0.5, 1.0, 5.0, and 10 mg C L^−1^). Three mL of 2 mM K-phosphate (pH 6.4) were added to each Petri dish to provide a growth medium. Cotyledons in Petri dishes were incubated at 27°C under continuous cool-white fluorescent light (10 µE m^−2^ s^−1^). Fresh weight measurements, for groups of five cotyledons, were made after blotting excess water. Percentage increases in fresh weight are expressed relative to initial fresh weight.

### Plant Material and Experimental Design

Garlic (*A. sativum* L., cv. Aglio Bianco Polesano) cloves were peeled and surface sterilized in 2–3% (v/v) H_2_O_2_ for 10 min. After being rinsed in deionized water, they were germinated in Petri dishes containing 1 mM CaSO_4_ solution, covered with aluminum foil and placed in the dark at 25°C for 4 d. Rooted cloves (plantlets) were then transferred inside glass cups (10 cloves per cup) containing 50 g of glass beads and 18 ml of Hoagland n. 2 solution (Hoagland and Arnon, 1950), and grown for 4 d with a 16 h light at 25°C and 60% relative humidity, 8 h of dark at 18°C and 80% relative humidity. At the end of this period, the plantlets were supplied for 48 h with a Hoagland n. 2 solution supplemented with HS (THE, HMS or LMS) at different concentrations (0.1, 0.25, 0.5, 1.0, 2.0, and 5.0 mg C L^−1^), or without HS (control). Plantlets were hence harvested and then carefully washed and dried with blotting paper. A sub‐sample of the root material was immediately frozen with liquid nitrogen and kept at −80°C to be used for biochemical analyses. For dry weight measurement, 10 plants per treatment from each pot were randomly harvested. The samples were placed in a drying oven for 2 d at 70°C and allowed to cool for 2 h inside a closed bell jar.

### Nitrate and Sulfate Quantification

Roots (1 g) developed from garlic plantlets were immersed in liquid N_2_ and homogenized in 10 mM HCl (1:5 w/v). The extract was filtered through two layers of muslin and clarified by centrifugation at 35,000 g for 15 min at 4°C. The supernatant was further filtered at 0.22 µm (Millipore), and the concentration of NO_3_^−^ and SO_4_^2−^ ions was determined using a High-Performance Liquid Chromatography (HPLC) system, through an AS 4S-SC anionic-exchange column (Dionex, Sunnyvale, CA), equipped with a Dionex suppressor and a 431 conductivity detector (Waters-Millipore, Milford, MA). A solution of sodium bicarbonate and sodium carbonate (1.7 mM NaHCO_3_/1.8 mM Na_2_CO_3_) was used as eluent at a flow rate of 2 mL min^−1^. Sodium nitrate and potassium sulfate were used as reference standards (Fluka, Buchs, Switzerland).

### Elemental Composition

Roots (0.5 g) of garlic plantlets were added with 9 mL of HNO_3_ (30% v/v) and H_2_O_2_ 30% (7:2), and placed in closed Teflon vessels of 100 mL volume. The digestion of root samples was carried out in a microwave system (Millestone Start-D 1200W). Mineralized samples were then diluted in 25 mL ultrapure water and the concentrations of Fe, K, Mg, and Ca were determined *via* inductively coupled plasma–atomic emission spectroscopy ICP-OES (Optima 2000 DV, Perkin Elmer Instruments Germany). Elements were quantified using certified multi-element standards.

### Enzyme Activities

Roots (1 g) of garlic plantlets were immersed in liquid N_2_ and homogenized (1:10 w/v) in 0.1 M potassium acetate buffer (pH 4.0) containing 0.1 M sucrose to determine invertase activity, or in 0.1 M phosphate buffer (pH 7.0) to test for peroxidase activity, or in 0.2 M Tris-HCl buffer (pH 7.0) to measure esterase activity. The extracts were centrifuged at 15,000 *g* for 15 min at 4°C and the supernatants were used as the enzyme source. Invertase activity was evaluated according to Arnold (1965), peroxidase activity as described by Putter (1974), esterase activity was determined as described by Junge and Klees (1984).

For nitrate reductase (NR), glutamine synthetase (GS) and O-acetylserine sulfhydrylase (OAS-s) measurements, enzymes were extracted from roots by manually crushing plant material in a mortar with a solution containing 100 mM HEPES (acido 4-2-idrossietil-1-piperazinil-etansolfonico)-NaOH (pH 7.5), 5 mM MgCl_2_ and 1 mM dithiothreitol (DTT). The ratio of plant material to mixture solution was 1:3 (w/v). The extracts were filtered through two layers of muslin and clarified by centrifugation at 20,000 *g* for 15 min. The supernatants were used as the enzyme sources. All steps were performed at 4°C. Nitrate reductase activity was assayed in a solution containing 100 mM KH_2_PO_4_, 100 mM KNO_3_, and the enzyme extract. The activity was measured spectrophotometrically at λ = 540 nm, and the calibration curve was plotted with known concentrations of NaNO_2_ (Lewis et al., 1982). With respect to the glutamine synthetase assay, the mixture contained 90 mM imidazole-HCl (pH 7.0), 60 mM hydroxylamine (neutralized), 20 mM KAsO_4_, 3 mM MnCl_2_, 0.4 mM ADP, 120 mM glutamine, and the enzyme extract. The assay was performed in a final volume of 750 μL. The enzymatic reaction was developed for 15 min at 37°C. The γ-glutamyl hydroxamate was determined by the addition of 250 μL of a mixture (1:1:1) of 10% (w/v) FeCl_3_ ·6H_2_O in 0.2 M HCl, 24% (w/v) trichloroacetic acid, and 50% (w/v) HCl. The optical density was recorded at λ =540 nm (Schiavon et al., 2008). O-acetylserine sulfhydrylase (OAS-s) activity was determined by measuring the production of L-cysteine, according to the procedure described by Kuske et al. (1994). Briefly, 1 mL reaction mixtures containing 100 mM Tris, 20 mM 0-acetylserine, 1 mM Na_2_S (pH 7.6) were initiated by addition of 1–10 μL of protein sample and further stopped by addition of 1.5 M trichloroacetic acid. Samples were centrifuged at 10,000 g for 5 min, and the amount of cysteine in the supernatants was estimated spectrophotometrically (λ =546 nm) using the ninhydrin reagent.

### Free Amino Acid Quantification

Free amino acids in roots were quantified according to Seebauer et al. (2004). Fifty mg of homogenous dry powder was extracted for 1 h at room temperature with 1.5 mL of a 5% (w/v) trichloroacetic acid (TCA) solution. The sample was clarified by centrifugation, and 1.5 mL of the supernatant was analyzed for free amino acids. The analysis of amino acid was realized using a precolumn OPA derivatization of the sample followed by reverse phase separation, through an Agilent 1100 HPLC (Agilent Technologies, Palo Alto, CA) equipped with a thermo-controlled auto-sampler, fluorescence detector and an Agilent HP Chemstation for data elaboration. The chromatographic conditions were described by Henderson et al. (2000). Aliin was extracted from roots of garlic and determined according to Hoppe et al. (1996).

### Light Microscopy

Two cm-long root tip of garlic plantlets grown with unfractionated (THE1-2) or size-fractionated (HMS1-2, LMS1-2) HS or not (control), were fixed with glutaraldehyde (6%) and processed for light microscopy as previously described (Bonghi et al., 1993). Cross thin sections (1 µm thick) of roots were cut with an Ultracut Reichert-Jung ultramicrotome, stained with 1% toluidine blue and 1% tetraborate (1:1, v/v), and observed and photographed under a Leitz Ortholux microscope.

### Statistical Analyses

Differences among mean values of chemical characteristics of HS were determined with the Student–Newman–Keuls test (*p ≤*0.05). The linear regression analysis (Y= a + bX) was used to verify the relationship between several chemical and biochemical parameters of garlic at different doses of unfractionated and size-fractionated HS. Prior to the regression analysis, the database was divided into two sub-samples based on HS concentration (0–1 mg C L^−1^ and 2-5 mg C L^−1^). Correlations between variables were determined using the Pearson’s coefficient.

Structure of the interdependences between unfractionated and size-fractionated HS chemical and biochemical parameters (C, H, N, O, S, COOH, phenolic-OH, aliphatic-C, peptidic and carbohydratic-C, aromatic-C, phenolic-C, carboxyl-C, IAA, IAA-like, GA-like, IPA-like) and plant physiological and biochemical attributes (root biomass, nitrate, sulfate, Ca, Mg, K and Fe content, nitrate reductase, glutamate synthetase, O-acetylserine sulphydrylase, invertase, peroxidase, esterase enzyme activities, and aspartate, threonine, isoleucine, lysine, asparagine, glutamate, serine, methionine, cysteine, aliin content) was performed using a joint Principal Components Analysis (PCA). The standardized variables were submitted to PCA; rotated orthogonal components (varimax rotation method) were extracted and the relative scores were determined. Only PCs with eigenvalue >1 were considered for the discussion. The object points were labeled by principal vegetation cover (1) *P. mugo* and (2) *P. sylvestris*, and molecular size of humic substances (total humic extracts, THE, and relative high, HMS, and low, LMS, molecular size fractions).

The Automatic Linear Modelling (ALM) was used to determine the factors which best influenced the hormone-like activities of unfractionated and size-fractionated HS. ALM was performed at the confidence level of 95%. All statistics were conducted using IBM SPSS Statistics for Windows version 25.

## Results

### Chemical-Spectroscopic Features, Hormone Content and Hormone-Like Activity of HS

The analysis of elemental composition and the spectroscopic characterization of total humic extracts (THE1-2) and relative high (HMS1-2) and low (LMS1-2) molecular size fractions indicated that some elements (C and N) and functional groups were unevenly distributed when THE1-2 were separated in size fractions during acetic acid treatment and dialysis procedure (**Table 1**). Precisely, THE1-2 were more enriched in C compared to HS fractions, but LMS1-2 were the highest in N. No significant differences were evident in O, H and S contents between HS. Oxygenated (carboxylic and phenolic-OH) groups were more abundantly present in LMS than in THE and HMS, with the maximum value reported for LMS2. Consistently, the 13C NMR integration areas (**Table 1**) showed greater occurrence of aromatic (105–145 ppm) and carboxylic (165-190 ppm) C in LMS1-2 (**Figure S1**), which were though the least abundant in protein and anomeric C (48–105 ppm) although this region may also include some signals of lignin and phenolic moieties (45–60 ppm) (Monda et al., 2017). The phenolic (145–165 ppm) C content, instead, did not vary significantly between THE and size-fractionated HS, except it was lower in LMS1. The hydrophobicity index and degree of aromaticity are usually used to indicate the biochemical and chemical stability of HS (Piccolo et al., 2005). The aromaticity of LMS1-2 was confirmed by the aromaticity degree (AD) values, which followed the trend THE1-2<HMS1-2<LMS1-2 (**Table 1**). Negative correlations (**Table S1**, Supplementary) were ascertained between the nominal size of HS and their aromatic degree (r = −0.72, *p* < 0.001), aromatic C (r = −0.65, *p* < 0.003), and carboxyl C (r = −0.65 *p* < 0.003). In contrast, positive correlations (**Table S1**, Supplementary) were found between the nominal size of HS and their carbohydratic-C (r = 0.54, *p* < 0.020), and aliphatic C (r = 0.77, *p* < 0.001). Evaluation of HS hydrophobicity based on HI/HB ratios (**Table 1**) indicated the elevated amount of aromatic C in LMS1-2, while the hydrophilic C was dominant in THE and HMS.

**Table 1:** Elemental composition, carboxylic and phenolic acidity and carbon distribution in 13C NMR spectra (ppm) of total humic extracts (THE) and its size-fractions (high molecular size, HMS and low molecular size, LMS) separated during acetic acid treatment and dialysis.

	Element					Acidity		13C NMR						
HS	C	H	N	O	S	COOH	Phenolic-OH	0-48	48-105	105-145	145-165	165-190	HI/HB^b^	AD^c^
	*%*					meq g^−1^		% Carbon distribution						
**THE1^a^**	58.1b^*^	4.2a	3.6c	36.1a	0.31a	5.2b	5.0b	27.3a	42.5a	15.6c	7.2a	7.4d	0.99	45.5
**HMS1**	56.2c	4.1a	2.8d	35.5a	0.15b	4.5b	5.5b	18.8b	44.1a	18.7b	7.4a	11.0c	1.22	58.1
**LMS1**	54.5d	4.5a	5.5b	32.2a	0.13b	6.2ab	5.5b	18.1b	20.2b	32.1a	5.2b	24.4b	0.80	67.3
**THE2**	59.3a	4.0a	3.8c	38.4a	0.28a	5.5b	6.0b	21.2b	42.4a	19.4b	7.0a	10.0c	1.10	55.4
**HMS2**	55.5d	3.9a	2.9d	34.6a	0.20ab	5.0b	5.8b	20.9b	43.2a	18.7b	6.8a	10.4c	1.15	54.9
**LMS2**	52.4e	4.2a	7.8a	33.1a	0.15b	7.5a	8.8a	14.8c	15.2c	33.7a	6.5a	29.8a	0.82	73.1

^a^(1) soil with Pinus mugo cover and (2) soil with Pinus sylvestris cover.

^b^HI/HB = hydrophobic index = [(48–105) + (165–190)]/[(0–48) + (105–165)].

^c^AD = aromaticity degree = (105–165)/(105–0)^*^ Values within column followed by the same label are not statistically different at p = 0.05 by Student-Newman-Keuls (Sokal and Rohlf, 1969).

The content of IAA (**Table 2**) varied significantly (*p* = 0.05) between THE, HMS and LMS, with a trend that was similar between HS from soils (1) and (2). LMS1-2 in particular, contained more IAA than THE1-2 and HMS1-2, with maximum values recorded in LMS2. Consistently with the trend of IAA content, the IAA-like activity (**Table 2**) was significantly higher (*p* ≤ 0.05) in LMS1-2, especially in LMS2. Similarly, the GA-like and IPA-like activities (**Table 2**) were prevalent in LMS1-2, mainly in LMS2, and were minimum in THE1-2.

**Table 2:** Indoleacetic acid (IAA) content and hormone-like activity (_†_) of total humic extracts (THE), high molecular size (HMS) and low molecular size (LMS) humic substances extracted from soil (1) (with *Pinus mugo* cover) and (2) (with *Pinus sylvestris* cover).

HS	IAA	IAA-like	GA-like	IPA-like
	% (w/w)	mg L^−1^ ≅ 1 mg C L^−1^		
**THE1**	0.01e^*^	0.14d	0.01e	0.25d
**HMS1**	0.03d	0.25d	0.04d	0.45b
**LMS1**	0.12b	1.67b	0.15b	0.84a
**THE2**	0.03d	0.72c	0.01e	0.12e
**HMS2**	0.05c	0.91c	0.07c	0.35c
**LMS2**	0.35a	5.25a	0.21a	0.79a

^†^Concentration (mg L^−1^) of indoleacetic acid (IAA) or gibberellic acid (GA) or isopentenyladenosine (IPA) of equivalent activity as 1 mg C L^−1^ humic substances.

^*^Values within column followed by the same label are not statistically different at p = 0.05 by Student-Newman-Keuls (Sokal and Rohlf, 1969).

### Effects of THE and Fractionated HS (HMS and LMS) on Garlic Plantlets

**Figure 1:**
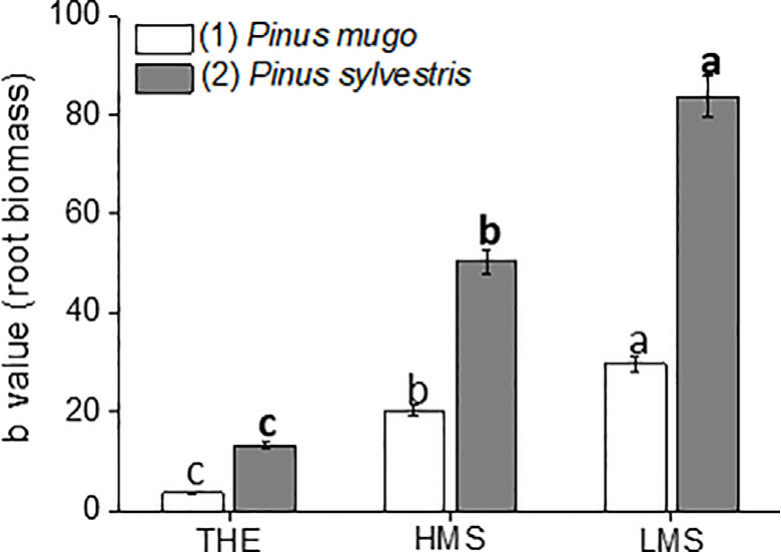
Root dry biomass of garlic plantlets grown in hydroponics and treated for 48 h with total humic extracts (THE) and size-fractionated HS (high molecular size, HMS and low molecular size, LMS) extracted from soil (1) (with *Pinus mugo* cover) and (2) (with *Pinus sylvestris* cover). The b values of the regression curve in the range 0–1mg C L^−1^ are reported. Different letters represent significant differences at *p* < 0.05 (n = 10). Unbolded letters compare HS from soil (1), letters in bold compare HS from soil (2).

To assay the dose-dependent effect of HS on garlic plantlet physiology and biochemical traits, a linear regression analysis of the whole data set was performed. Linear regression analyses were all significant (R^2^ ranging from 0.60 to 0.99, *p* ≤ 0.01) in terms of independent variables (**Supplementary Tables S2**-**S4**). Despite a big amount of data was generated from this analysis, we focused on the description of the most relevant findings. Thus, the regression coefficient (b) ± SE values of each curve, calculated in the HS concentration range of 0.1–1.0 mg C L^−1^ (linear positive range, first trait), were shown in **Figures 1** **–** **4**. The b value was indicative of how the HS dose and type (THE, HMS, and LMS) impacted on plant growth and biochemical attributes. Comparisons for statistical differences were accomplished within the same group of HS, i.e. HS derived from soil (1) or soil (2). Data referred to higher doses of HS (2 and 5 mg C L^−1^, second trait) are reported in **Tables S2**-**S4** (Supplementary). In this case, less effect or no effect of HS on all plant traits subjected to evaluation was observed.

**Figure 2:**
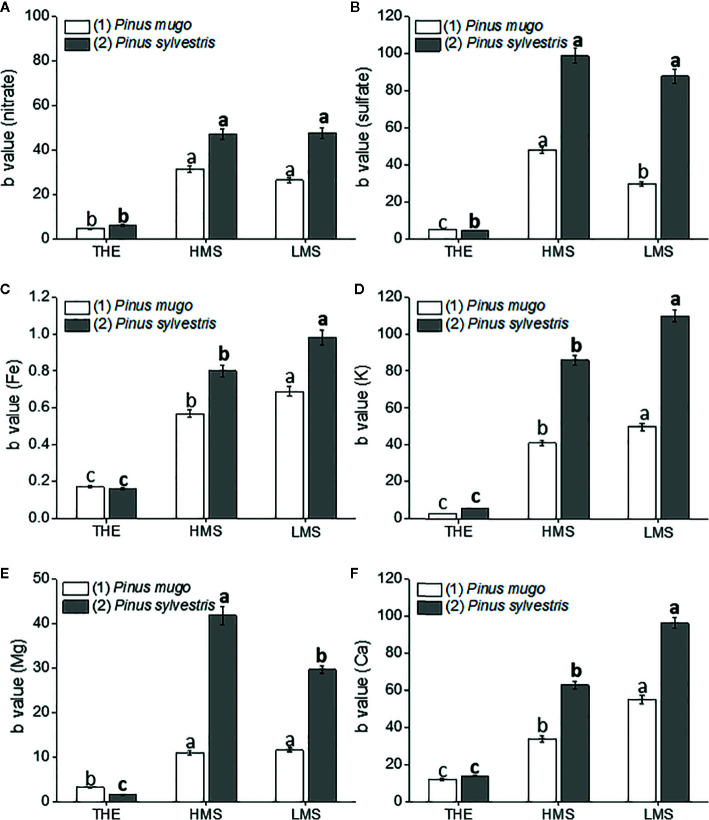
Root nutrient [nitrate **(A)**, sulfate **(B)**, Fe **(C)**, K **(D)**, Mg **(E)**, Ca **(F)**] content of garlic plantlets grown in hydroponics and treated for 48 h with total humic extracts (THE) and size-fractionated HS (high molecular size, HMS and low molecular size, LMS) extracted from soil (1) (with *Pinus mugo* cover) and (2) (with *Pinus sylvestris* cover). The b values of the regression curve in the range 0 –1mg C L^−1^ are reported. Different letters represent significant differences at *p* < 0.05 (n = 5). Letters not in bold compare HS from soil (1), letters in bold compare HS from soil (2).

**Figure 3:**
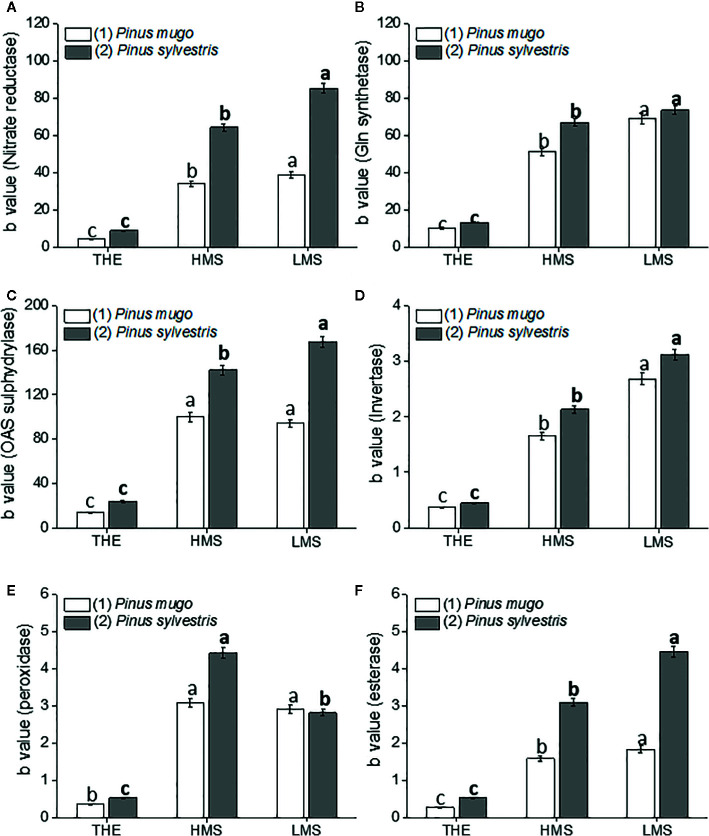
Activity of nitrate reductase **(A)**, glutamine synthetase **(B)**, O-acetylserine sulphydrylase **(C)**, invertase **(D)**, peroxidase **(E)**, esterase **(F)** enzymes in roots of garlic plantlets grown in hydroponics and treated for 48 h with total humic extracts (THE) and size-fractionated HS (high molecular size, HMS and low molecular size, LMS) extracted from soil (1) (with *Pinus mugo* cover) and (2) (with *Pinus sylvestris* cover). The b values of the regression curve in the range 0 –1mg C L^−1^ are reported. Different letters represent significant differences at *p* < 0.05 (n = 5). Letters not in bold compare HS from soil (1), letters in bold compare HS from soil (2).

**Figure 4:**
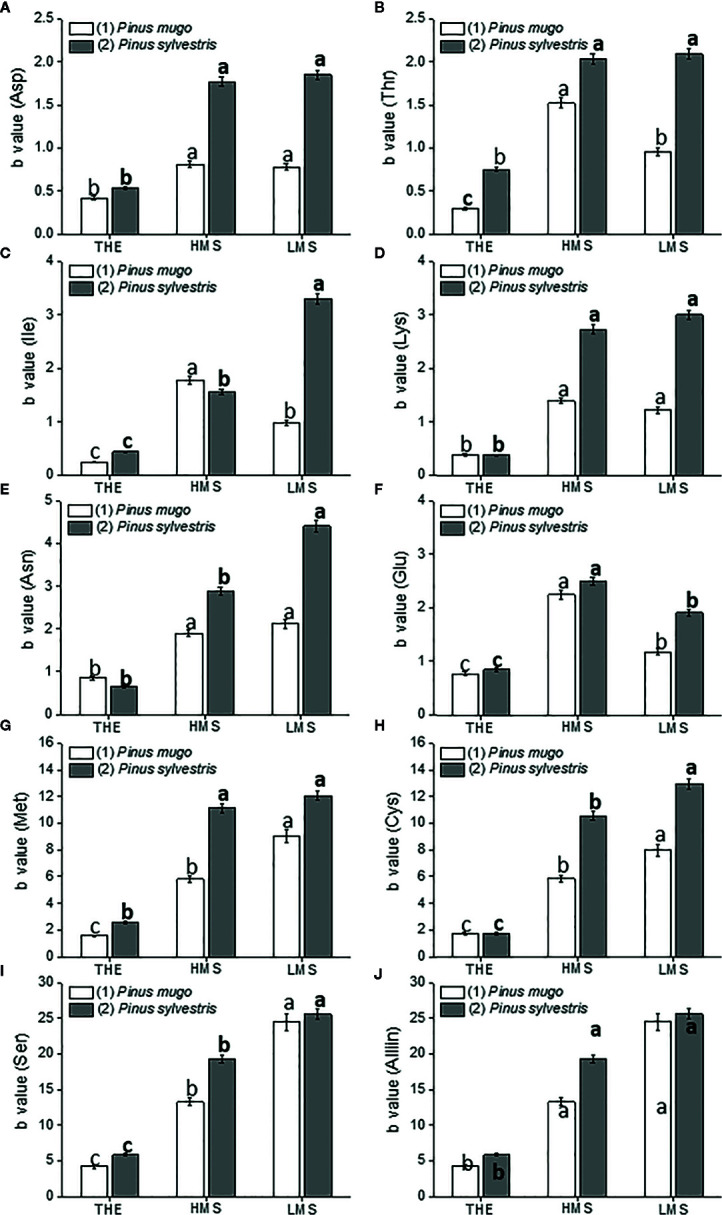
Amino acid content in roots of garlic plantlets grown in hydroponics and treated for 48 h with total humic extracts (THE) and size-fractionated HS (high molecular size, HMS and low molecular size, LMS) extracted from soil (1) (with *Pinus mugo* cover) and (2) (with *Pinus sylvestris* cover). The b values of the regression curve in the range 0 –1mg C L^−1^ are reported. Panels are: **(A)** Asp, asparagine; **(B)** Thr, threonine; **(C)** Ile, isoleucine; **(D)** Lys, lysine; **(E)** Asn, asparagine; **(F)** Glu, glutamate; **(G)** Met, methionine; **(H)** Cys, cysteine; **(I)** Ser, serine; **(J)** Aliin. Different letters represent significant differences at *p* < 0.05 (n = 5). Letters not in bold compare HS from soil (1), letters in bold compare HS from soil (2).

### Effects of HS on Root Growth and Nutrient Content of Garlic Plantlets

The root biomass showed considerable variation depending on HS treatment (**Figure 1**, **Supplementary Table S2**). The trends observed for root biomass of plantlets treated with HS from either soil (1) or (2) were similar, although values were generally higher when HS derived from soil (2). Maximum and minimum root growth was ascribed to plantlets receiving LMS1-2 and THE1-2, respectively, with differences becoming more evident when HS fractions were extracted from soil (2). Indeed, LMS2-treated plantlets produced more root biomass than LMS1-treated plants (+66%) when compared to plantlets supplemented with HMS2 or HMS1 (+46%).

Garlic plantlets accumulated more nutrients (nitrate, sulfate, Fe, K, Mg, and Ca) when treated with HS fractions from soil (2) (**Figures 2A–F**). HMS2 and LMS2 induced similar accumulation of nitrate (NO_3_^−^) and sulfate (SO_4_^2−^) ions, while THE2 determined the lowest increments (**Figures 2A, B**). Similar findings were reported in plantlets supplemented with HS from soil (1). In this case, however, the sulfate content in plantlets subjected to HMS1 treatment was higher than in plantlets added with LMS1. The b values of the regression curves that refer to the root content of other mineral nutrients (Fe, K, and Ca) revealed a similar trend for the effects of HS from the two soils: THE<HMS<LMS (**Figures 2C, D, F**). Unlike Fe, K, and Ca, Mg accumulated more in HMS2- than in LMS2-treated plantlets, while no differences were evident between plantlets supplied with HS fractions from soil (1) (**Figure 2E**). As for the other elements, Mg accumulated less in roots of plantlets treated with THE.

### Effects of HS on Plant Metabolism and Amino Acid Content at the Root Level

Treating garlic plantlets with HS prompted differential effects on enzymes involved in N metabolism (nitrate reductase and glutamine synthetase), S assimilation (OAS-sulphydrylase), and developmental processes (invertase, peroxidase and esterase) (**Figures 3A–F**). Such distinct effects mainly relied on HS type and soil of origin. HMS1-2 and LMS1-2 stimulated the activity of tested enzymes more than THE1-2, thus suggesting that THE1-2 was less effective than size-fractionated HS to enhance the primary metabolism and growth of garlic. The activities of all enzymes, except peroxidase, behaved similarly when plantlets were supplied with HS fractions from soil (2), as higher stimulation was generally induced by LMS2 over HMS2. HS fractions from soil (1) triggered similar increments in activity of nitrate reductase, OAS-sulphydrylase, peroxidase and esterase enzymes, but LMS1 intensified the activity of glutamine synthetase and invertase enzymes more than HMS1. The activity of the enzymes nitrate reductase, OAS-sulphydrylase and esterase was more increased in garlic roots after treatment with HS from soil (2). For the other enzymes, the stimulatory effect of their activity was comparable between HS from soil (1) and soil (2).

Maximum accumulation of amino acids, with the exception of glutamate (Glu), was evident in garlic roots treated with LMS2 (**Figures 4 A–J**). THE1-2 instead, was the least effective in increasing the content of all amino acids, while the effect of HS fractions from soil (1) varied depending on the target amino acid. HMS1 and LMS1 induced similar accumulation of aspartate (Asp), lysine (Lys), asparagine (Asn) and Alliin. HMS1, though, triggered higher accumulation of threonine (Thr), isoleucine (Ile) and Glu. Conversely, S amino acids (methionine, Met, cysteine, Cys), and serine (Ser) accumulated more under LMS1 treatment. HS fractions from soil (2) were however more effective than HS fractions from soil (1) in promoting the accumulation of several amino acids (Asp, Thr, Lys, Asn, Met, Cys, Alliin). Also, HMS2 and LMS2 provoked comparable incremental accumulation of Asp, Thr, Lys, Met and Alliin, while LMS2 induced higher accumulation of Ile, Asn, Cys and Ser. Glu was the only amino acid that accumulated more under HMS2 treatment.

### Effects of HS on Root Anatomy

**Figure 5:**
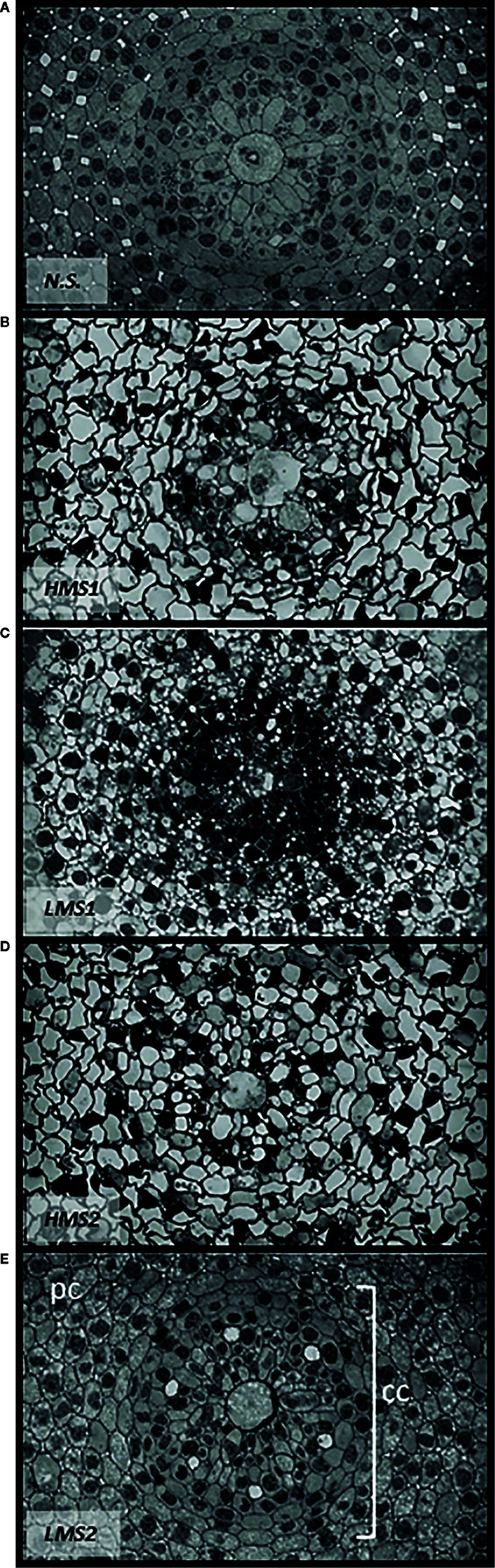
Root anatomy of garlic plantlets grown in hydroponics with nutrient solution **(A)** (SN) without HS, or treated for 48 h with size-fractionated HS **(B, D)** high molecular size, HMS and **(C**, **E)** low molecular size, LMS) extracted from soil (1) (with *Pinus mugo* cover) and (2) (with *Pinus sylvestris* cover). CC, central cylinder; PC, parenchyma cells.

The analysis of root anatomy emphasized differences in cell differentiation between garlic plantlets grown in nutrient solution without HS (control) and plantlets treated with either HMS1-2 or LMS1-2 (**Figures 5A–E**). LMS1-2 caused a more evident and earlier differentiation pattern in the central cylinder (**Figures 5C, E**), especially with respect to protoxylem vessel formation, compared to HMS1-2 (**Figures 5B, D**) and the nutrient solution solely (**Figure 5A**). The differentiation process was more pronounced in the root central cylinder of garlic plantlets treated with LMS derived from soil (2). However, HMS1-2 induced higher differentiation of cortical cells, which appeared bigger in size and contained a unique vacuole per cell. The root parenchyma of control plantlets was formed by cells with no vacuoles, therefore late in differentiation. In contrast, plantlets supplied with LMS showed a differentiation process in progress, as cells showed substantial vacuolization.

### Correlation Analysis

The Pearson’s correlation analysis (**Table S1**, Supplementary) highlighted significant relationships between quantitative variables of HS, as well as between metabolic traits of garlic plantlets after treatment with HS. In most cases, the r value ranged from 0.77 to 0.77–0.99 (*p* ≤ 0.01). As an example, Alliin greatly correlated with Cys (r = 0.97, *p* ≤ 0.01) and Lys (r = 0.96, *p* ≤ 0.01), while the IPA-like activity negatively correlated with O (r = −0.93, *p* ≤ 0.01).

### Relationship Between Hormone-Like Activity of HS and Plant Biochemical Attributes Assayed by Automatic Linear Modeling

The ALM allowed to get the outcome of three linear models explaining the relationship between the hormone-like activity of HS and the biochemical attributes of garlic plantlets (**Table 3**). All calculated models provided high accuracy, which was equal to 99.7, 99.85, and 100% for GA-like, IPA-like and IAA-like activity, respectively. The IAA-like activity was explained by 7 variables over 21 assayed variables. The relative importance (i) and significance of the obtained predictive variables followed the order N (i = 0.442, *p* ≤ 0.000) > OAS-sulfhydrylase activity (i = 0.292, *p* ≤ 0.000) > S (i = 0.175, *p* ≤ 0.000) > IAA (i = 0.042, *p* ≤ 0.002) > phenolic-OH (i = 0.020, *p* ≤ 0.016) > ISO (i = 0.016, *p* ≤ 0.027) > O (i = 0.013, *p* ≤ 0.045). All the 7 variables entering the equation positively influenced the IAA-like activity target variable. The GA-like activity model was explained by such variables as sulfate (i = 0.475, *p* ≤ 0.000), carboxyl-C (i = 0.339, *p* ≤ 0.000), OAS-sulphydrylase activity (i = −0.163, *p* ≤ 0.001) and peptidic-C (i = −0.024, *p* ≤ 0.109). The first two predictive variables entered the equation with a positive effect on GA-like activity, while the other two predictive variables exerted a negative effect. For the IPA-like activity, the model was explained by Mg (i = −0.368, *p* ≤ 0.000), peptidic-C (i = −0.284, *p* ≤ 0.000) and O (i = −0.251, *p* ≤ 0.000), which displayed a negative effect, and by Cys (i = 0.079, *p* ≤ 0.000), Fe (i = 0.009, *p* ≤ 0.017) and phenolic-C (i = 0.009, *p* ≤ 0.021) which conversely determined a positive effect.

**Table 3:** Predictive importance (i) of chemical and biochemical variables to hormone-like (IAA-like, GA-like, IPA-like) activity of humic substances by Automatic Linear Modeling.

	Target variable							
	IAA-like		GA-like			IPA-like		
Predictor	i	*p* value	Predictor	i	*p* value	Predictor	i	*p* value
**N**	0.442	0.000	**SO_4_^2-^**	0.475	0.000	**Mg**	−0.368	0.000
**OAS-s**	0.292	0.000	**Carboxyl-C**	0.339	0.000	**Peptidic-C**	−0.284	0.000
**S**	0.175	0.000	**OAS-s**	−0.163	0.001	**O**	−0.251	0.000
**IAA**	0.042	0.002	**Peptidic-C**	−0.024	0.109	**Cys**	0.079	0.000
**Phenolic-OH**	0.020	0.016				**Fe**	0.009	0.017
**Ile**	0.016	0.027				**Phenolic-C**	0.009	0.021
**O**	0.013	0.045						

Carboxyl-C, Carboxyl-C in HS by NMR; Cys, cysteine content in plants roots; Fe, iron content in plants roots; IAA, indoleacetic acid content in HS by ELISA; Ile, isoleucine content in plants roots; Mg, magnesium content in plants roots; N, nitrogen in HS by elemental composition; O, oxygen in HS by elemental composition; OAS-s, O-acetylserine sulphydrylase activity in plants roots; Peptidic-C, Peptidic-C in HS by NMR; Phenolic-C, Phenolic-C in HS by NMR; Phenolic-OH, Phenolic-OH acidity in HS by titration; S, sulfur in HS by elemental composition; SO_4_^2−^, sulfate content in plants root.

### Principal Component Analysis

The PCA analysis revealed that three factors accounted for 95.2% of the total variance. Principal component 1 (PC1) explained 53.9% of the variance, and positively correlated with OAS-sulphydrylase activity, Cys, Lys, Mg, sulfate, esterase activity, glutamate synthetase, nitrate, invertase, Alliin, Ca, K, Met, Ser, peroxidase activity, nitrate reductase activity, Glu, Fe, Asp, Thr, Asn, Ile, and root biomass (**Table S5**, Supplementary). Principal component 2 (PC2) explained 31.2% of the total variance and was positively correlated with carboxyl-C, N, IAA-like, IAA, phenolic-OH, GA-like, COOH, aromatic-C, and negatively with aliphatic-C, C and peptidic-C (**Supplementary Table S5**). The remaining 10% of the total variance was explained by Principal component 3 (PC3), which mostly correlated with phenolic-C, H, S, and O (**Supplementary Table S4**).

**Figure 6:**
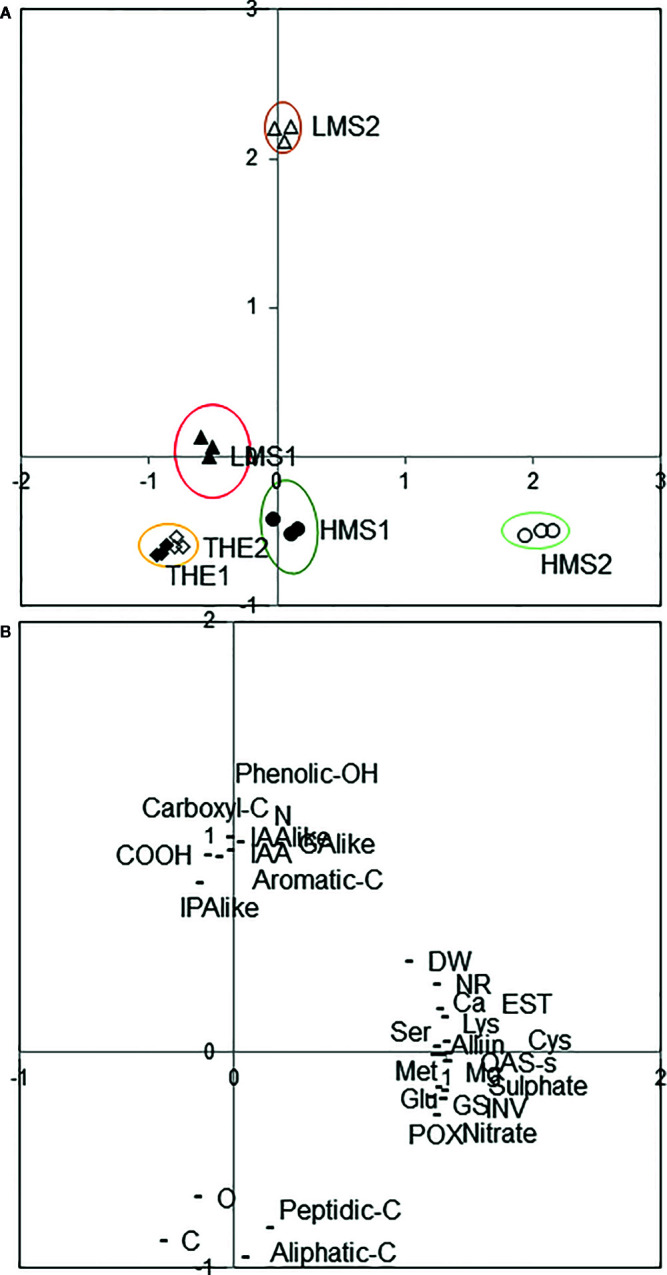
Positions of the unfractionated (THE, total humic extract) and fractionated humic substances (HMS, high molecular size; LMS, low molecular size) (1) with *Pinus mugo* cover, (2) with *Pinus sylvestris* cover in the reduced space of the ﬁrst two principal components after principal components analysis **(A)**. Variables projected in the plane determined by the ﬁrst two principal axes **(B)**: HS chemical and biochemical parameters (aliphatic-C, aromatic-C, C, carbon, carboxyl-C, COOH, GA-like, gibberellin-like activity, IAA, indoleacetic acid content, IAA-like, auxin-like activity, IPA-like, cytokinin-like activity, N, nitrogen, peptidic and carbohydratic-C, phenolic-OH, O, oxigen), and plant physiological and biochemical attributes (Alliin; Ca, calcium; Cys, cysteine; DW, root dry weight; EST, esterase activity; Glu, glutamate; GS, glutamine synthetase activity; INV, invertase activity; Lys, lysine; Mg, magnesium; Met, methionine; NR, nitrate reductase activity; OAS-s, O-acetylserine sulphydrilase activity; POX, peroxidase activity; Ser, serine).

Plotting data according to PC1 and PC2, treatments resulted in well separated HS of soil (1) from HS of soil (2), and HMS versus LMS, while THE scattered around the origin (**Figure 6A**). In particular (**Figure 6B**), variables related to plant biochemical traits distinguished HMS2 from HMS1, while variables related to HS characteristics differentiated LMS2 from LMS1. Notably, HMS2 was characterized by the highest values in OAS-sulphydrylase activity, Cys, Lys, Mg, sulfate, esterase activity, glutamate synthetase activity, nitrate, invertase activity, Alliin, Ca, K, and Met, while LMS2 by the highest values in carboxyl-C, N, IAA-like, IAA, GA-like, IPA-like and phenolic-OH.

## Discussion

Plant responses to humic substances depend on HS nature, molecular size, chemical properties and concentration (Canellas and Olivares, 2014). Our results proved the impact of the plant cover at the sampling soil on the formation of bioactive HS with distinctive chemical-structural features. The two sampled soils were similar in properties, but differed in plant cover. From a previous study (Nardi et al., 2000) we know that *P. mugo* litter on soil (1) was characterized by a high content of lignin, proteins and ash, while *P.* s*ylvestris* litter on soil (2) contained a large amount of hemicellulose, cellulose, fats, waxes and oils. The effect of the litter composition on HS chemistry and bioactivity was more evident for LMS1-2, as these fractions hold a higher content of N and oxygenated (-COOH and OH-phenol) functional groups, and displayed greater aromaticity compared to THE1-2 and HMS1-2. Interestingly, such parameters were all maximally intensified in LMS2.

The molecular fractionation method used in this study was based on HS treatment with a weak monocarboxylic acid, namely acetic acid. Such a treatment reduced the complexity of HS (Nardi et al., 1988) by leading to a progressive disturbance of the supramolecular humic structures resulting from the association of different chemical species (Piccolo, 2001; Piccolo, 2002). Although the disassociation induced by acetic acid relies on the nature of the associated species, it might overcome the properties of the isolated components, as a result of the specific molecular interactions controlling the affinity, organization and cooperation between aggregates. In this context, new molecular rearrangements could be responsible for diverse pathways of HS molecular communication with plants (Canellas et al., 2019). In general, the action exerted by acetic acid is comparable to that produced by root exudation or microbial transformation of organic matter (Nardi et al., 1997). Both these processes can promote the release of bioactive molecules that trigger positive effects in plants and the rhizosphere (Nardi et al., 2002; Nardi et al., 2005; Nardi et al., 2017).

The formation of simpler aggregates differing in size (HMS and LMS) defines differences in bioactivity between them and compared to unfractionated HS, because of their typical chemical composition and spatial distribution of hydrophilic and hydrophobic domains (Nebbioso and Piccolo, 2012). Previously, Nardi et al. (2007) showed that LMS was endowed with high hydrophilic degree due to the predominant carbohydrate component, which accounted for the greater bioactivity of this fraction. In this respect, LMS has been hypothesized to assume a specific spatial arrangement responsible for better interaction with the plant roots. However, other studies (Canellas et al., 2008; Canellas et al., 2009; Canellas et al., 2010) highlighted a strong relationship between the hydrophobic domain of HS and the H^+^-ATPase activity of plant roots. In that case, HS bioactivity was ascribed to the capacity of the hydrophobic domain to promote the release of biologically active molecules, such as auxin-like substances, that target the plasma membrane H^+^-ATPases (Canellas et al., 2010). Such effect was reported to be sparked by the action of root exudates (Canellas et al., 2019). Our results clearly show that the elevate bioactivity of LMS1-2 was heavily dependent on their high aromaticity degree and content of polar residues, while the hydrophobic domain likely contributed to the stability and functionality of LMS aggregates (Monda et al., 2018). This rationale is supported by the highest values of IAA content and hormone-like activities measured in LMS1-2. The hydrophilic domain was apparently less important in dictating the structure of THE and HMS because it did not contribute to the activity of HS appreciably. In this respect, the hydrophobic/hydrophilic ratio of a humic aggregate may be indicative of its bioactivity potential.

A clear relationship between HS dose and early effects on biochemical attributes of garlic plantlets was also determined in this study within a short-period (48 h) of plantlet treatment with HS. This short period has long been used to evaluate the biostimulant activity of HS and other products on hasty changes in plant metabolism (Schiavon et al., 2008; Schiavon et al., 2010; Ertani et al., 2018; Schiavon et al., 2019). To estimate the dose-response function, several concentrations of unfractionated and size-fractionated HS were tested, and fitting curves were build-up. A linear regression model described the behavior of HS, which was positive at low HS doses, while negative at high HS doses. More specifically, the beneficial effects of HS on plant growth, nutrition and metabolism were evident in a close range of HS concentration, i.e., from 0.1 to 1 mg C L^−1^, regardless of HS type (unfractionated or size-fractionated). Higher HS doses produced less or no effect.

Within the positive linear regression range, the most remarkable effects on garlic root growth and physiology were elicited by LMS1-2, thereby confirming the high bioactivity of these HS fractions inferred by the primary chemical-structural analyses. The elevate IAA content and hormone-like activity of LMS1-2 conceivably justified the capacity of these two fractions to induce the highest increase in root biomass and earliest root cell differentiation patterns in the central cylinder. The IAA-like activity strongly correlated with the IAA content, but could additionally be ascribed to other auxins (e.g., phenylacetic acid and indole butyric acid) (Russell et al., 2006) and aromatic biologically active compounds, such as phenol-C groups (Muscolo et al., 2007). The phenol-C groups can also be responsible for the GA-like and IPA-like activities of HS (**Table 3**). Auxins and auxin-like substances are tightly connected with the root development by controlling the increase in length of root hairs, the primary root length, and the number of lateral root primordia (Overvoorde et al., 2010), while the activity of GA principally targets the endodermis to regulate root growth and cell elongation (Ubeda-Tomás et al., 2008). Consistently with our findings, previous studies reported that root development is positively affected by the activity of hormones and hormone-like substances that are mediated or released by HS nearby the root system (Mora et al., 2010; Zanin et al., 2019). With respect to the IPA-like activity, it must be noted that cytokinins are positive plant growth regulators (Müller and Sheen, 2007; Müller and Sheen, 2008). However, at root level they can exert antagonistic effects on auxin-mediated responses by altering the expression of IAA transporters (PIN) in lateral root founder cells, thus preventing the formation of the auxin gradient required to shape lateral root primordia (Laplaze et al., 2007). Interestingly, the ratio IAA-like to IPA-like activity of LMS1-2 was about 3.6 fold higher compared to that of THE1-2 and HMS1-2, which may suggest that the maximum increase of root biomass observed in LMS-treated plantlets was at least in part due to less antagonistic inhibition exerted by IPA-like activity. Also, when the ratios IAA-like to IPA-like activity were compared between HS derived from the two soils, values were higher for HS from soil (2). These ratios are coherent with the observation that HS derived from soil (2) stimulated root growth more than HS from soil (1) (Nardi et al., 2000), as indicated by the higher b values of the regression curve (**Table S1**, Supplementary).

The trend of root growth was wholly consistent with the activity of the enzymes invertase and esterase, which have established roles in plant development and proved to be activated by plant biostimulants (Ertani et al., 2018). The enzyme esterase is implied in organogenesis processes and represents an early gauge of somatic embryogenesis (Balen et al., 2004), while the enzyme invertase controls plant growth by regulating the availability of hexose substrates for cellular metabolism, especially in sinks experiencing cell expansion (Tauzin and Giardina, 2014). Peroxidase activity, which is also involved in cellular differentiation processes (Balen et al., 2004), performed differently from esterase and invertase activities being preferentially targeted by HMS2. However, it was similarly more induced by size-fractionated HS from soil (2).

The hexose sugars released by sucrose hydrolysis can also be used to drive other energy-dependent processes, including the active transport of mineral nutrients. In our study, we measured higher accumulation of nitrate and sulfate ions in roots of garlic plantlets when treated with sized-fractionated HS. Nevertheless, no differences in nitrate were observed between plantlets treated with LMS and HMS derived from the same soil, while sulfate accumulation tended to decrease in LMS treated-plantlets. A possible explanation is that nitrate and sulfate ions were more consumed in plantlets receiving LMS because of higher assimilation rates, as suggested by higher activity of N (nitrate reductase and glutamine synthetase) and S (OAS-sulphydrilase) assimilation enzymes, and increased content of certain amino acids (e.g., Ile, Asn, Ser, Cys). So far, many studies have reported the stimulation of N metabolism in plants by either HS or other biostimulants (Schiavon et al., 2008; Santi et al., 2017; Palumbo et al., 2018; Zanin et al., 2018; Schiavon et al., 2019), while scarce literature exists on the effects of HS on the S pathway. Jannin et al. (2012) in particular, showed that treating *Brassica napus* plants with HS positively impacted on C, N and S metabolism, as the expression of several genes implied in primary metabolic pathways and in N and S uptake was substantially upregulated. We found that cysteine and its precursor serine accumulated more in roots of LMS-treated garlic plantlets. Cysteine in turn serves as a substrate for the synthesis of other S amino acids, i.e. methionine and the non-proteinogenic amino alliin (S-allylcysteine sulfoxide). Accumulation of S amino acids was significantly higher when size-fractionated HS were applied to garlic, especially if they were derived from soil (2). The high increase of alliin content in LMS2- and HMS2-treated plantlets is a valuable finding that might have relevant implications for the nutritional quality of garlic. Indeed, upon garlic clove tissue damage, the compound allicin is produced from alliin in a reaction catalyzed by the enzyme alliinase. Allicin displays a number of health-promoting properties by acting as a powerful antimicrobial, antifungal and anticarcinogenic agent, and by lowering cholesterol and blood pressure with benefits for the cardio-vascular system (Borlinghaus et al., 2014). Intriguingly, the hormonal-like activity of HS, either from soil (1) or soil (2), was toughly linked to S metabolism (**Table 3**), thus confirming the relationships between hormones and S nutrition described in other studies (Dan et al., 2007; Falkenberg et al., 2008; Masood et al., 2016; Hasanuzzaman et al., 2018).

Similarly to nitrate and sulfate, the elements K, Fe, Mg and Ca accumulated more in garlic roots when plantlets were treated with sized-fractionated HS than with total HS extracts (THE), which reflected the greater capacity of LMS and HMS to induce more positive effects on plant nutrition. With the exception of Mg, the other nutrient elements followed the same trend as root growth. The increased accumulation of nutrient elements by HS and other biostimulants has been widely reported in crops, including garlic (Denre et al., 2014). HS can improve plant nutrition through direct and indirect mechanisms (Zandonadi et al., 2010; Shah et al., 2018), including the enhancement of root plasma membrane H^+^-ATPase activity *via* hormones and NO signaling pathways, the increase of micro- and macro- nutrient bioavailability *via* formation of soluble ions-HS complexes (Garcia-Mina et al., 2004; Olaetxea et al., 2019), targeted and non-targeted effects at the cells membranes that trigger biochemical and molecular cascade transcriptional and post-transcriptional events regulating the expression of nutrient transporters (Van Oosten et al., 2017).

Based on the PCA analysis, it seems evident that the physiological effects elicited by LMS and HMS differed depending on the chemical properties and origin of HS. Interestingly, THE1 and THE2 were comparable in features and displayed similar capacity to influence plant metabolism, thus confirming the efficacy of weak organic acids to produce size-fractionated HS more bioactive than unfractionated HS.

## Conclusions

In conclusion, this study confirms that importance of the cover vegetation in determining the bioactivity of HS. Treating THE with a weak acid produced sized-fractionated HS that exhibited different chemical properties and bioactivity, likely because of novel molecular arrangements that better interacted with the plant roots. Size-fractionated HS from forest soils were more bioactive than unfractionated HS, but LMS1-2 were the most effective in improving the biochemical and physiological attributes of garlic over 48 h owing to their chemical-structural properties. We also show that the bioactivity of LMS1-2 was heavily influenced by the elevated aromaticity, the large content of polar residues and the more pronounced IAA content and hormone-like activity. Another important finding of this study is that size-fractionated HS greatly stimulated S metabolism beside N assimilation, with positive implications for the nutritional value of this crop and human health. Although treating plants with HS for a short period has long been used to test their biostimulant properties, to reinforce the results obtained in this study and to verify the effects of HS on garlic productivity, a pot study conducted for a longer period until garlic harvest would be needed in the future.

## Data Availability Statement

The raw data supporting the conclusions of this article will be made available by the authors, without undue reservation.

## Author Contributions

SN conceived and designed the experiments and contributed to data interpretation. DP contributed to data acquisition, analysis, and interpretation. OF and MS contributed to data analysis and interpretation. FDV contributed to data acquisition, analysis, and interpretation for the root ultrastructure. AE contributed to data interpretation. DP, SN, OF, MS, FDV, and AE drafted the manuscript. MS, DP, OF, and SN were the major contributor to the writing of the manuscript.

## Funding

This research was founded by DOR 2019 (SN) provided by University of Padova.

## Conflict of Interest

The authors declare that the research was conducted in the absence of any commercial or financial relationships that could be construed as a potential conflict of interest.

## Supplementary Material

Figure S1 13C-NMR spectra of low molecular size LMS humic substances extracted from soil (1) (with *Pinus mugo* cover) and (2) (with *P. sylvestris* cover).

Table S1 Correlation matrix (Pearson) for the chemical and biochemical variables.

Table S2 Parameters of the regression curves (first and second traits) between concentration of humic substances (total humic extract, THE, high molecular size, HMS and low molecular size, LMS) and root biomass and macro and micro nutrients content in HS-treated garlic plantlets.

Table S3 Parameters of the regression curves (first and second traits) between concentration of humic substances (total humic extract, THE, high molecular size, HMS and low molecular size, LMS) and enzyme activities in roots of HS-treated garlic plantlets.

Table S4 Parameters of the regression curves (first and second traits) between concentration of humic substances (total humic extract, THE, high molecular size, HMS and low molecular size, LMS) and amino acid content in roots of HS-treated garlic plants.

Table S5 Loadings values of chemical and biochemical variables on the axes identified by principal components analysis.
