# Supplementary figures and images for: Bioactivity of Size-Fractionated and Unfractionated Humic Substances From Two Forest Soils and Comparative Effects on N and S Metabolism, Nutrition, and Root Anatomy of Allium sativum L
Source: Front Plant Sci. 2020 Aug 14;11:1203. doi: 10.3389/fpls.2020.01203 (PMC7457123; doi:10.3389/fpls.2020.01203)

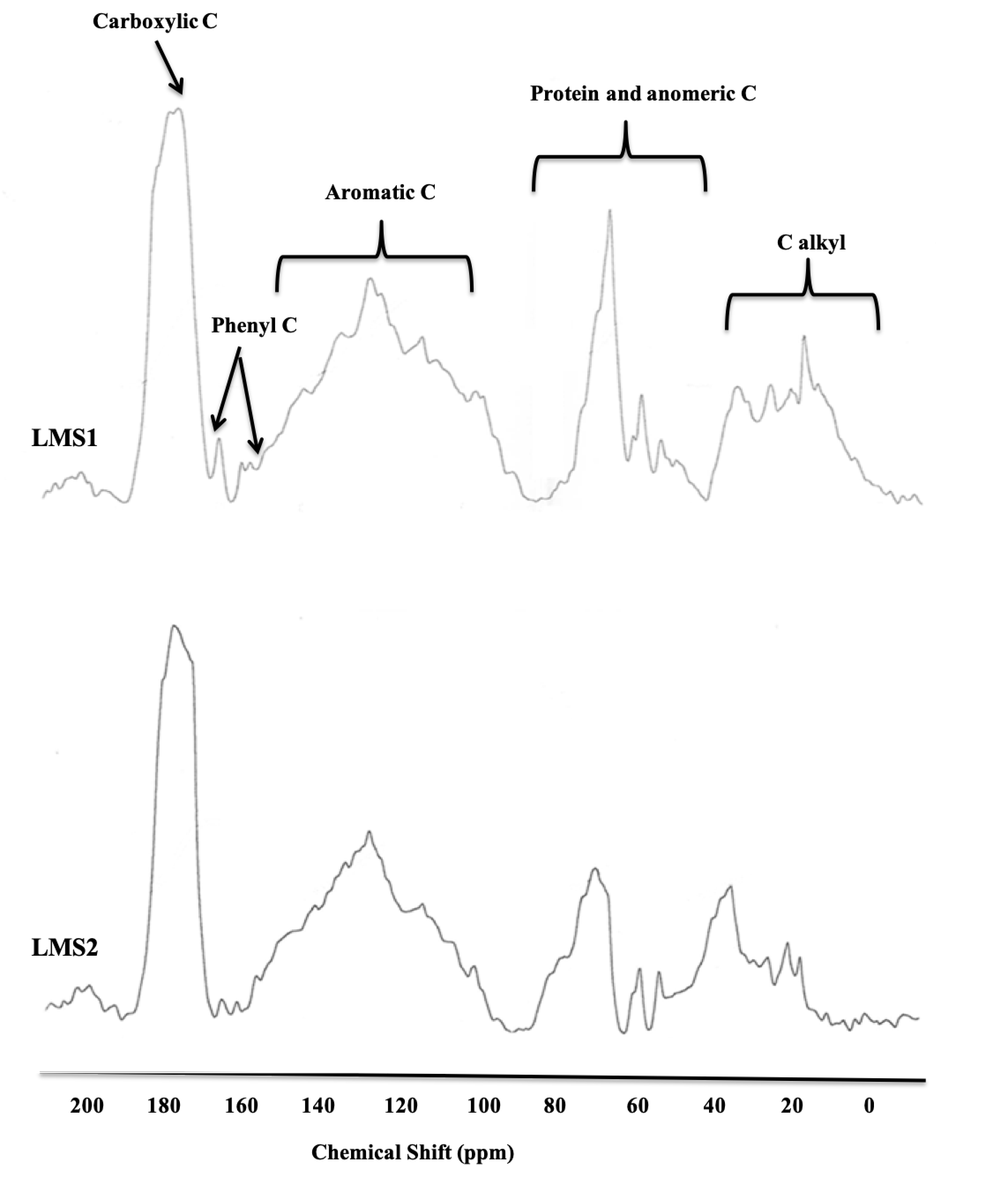

Supplement: Figure S1 — 13C-NMR spectra of low molecular size LMS humic substances extracted from soil (1) (with Pinus mugo cover) and (2) (with P. sylvestris cover). [file Image_1.tif]
