# Supplementary material for: Bioactivity of Size-Fractionated and Unfractionated Humic Substances From Two Forest Soils and Comparative Effects on N and S Metabolism, Nutrition, and Root Anatomy of Allium sativum L
Source: Front Plant Sci. 2020 Aug 14;11:1203. doi: 10.3389/fpls.2020.01203 (PMC7457123; doi:10.3389/fpls.2020.01203)
Supplement: Table S1 — Correlation matrix (Pearson) for the chemical and biochemical variables. [file Table_1.docx]

**Supplementary Material**

**Bioactivity of size-fractioned and unfractionated humic substances from two forest soils and comparative effects on N and S metabolism, nutrition and root anatomy of *Allium sativum* L.**

**Diego Pizzeghello^1*^, Michela Schiavon^1^, Ornella Francioso^2^, Francesca Dalla Vecchia^3^, Andrea Ertani^4^, Serenella Nardi^1^**

*** Correspondence:** diego.pizzeghello@unipd.it

**Table S1** Correlation matrix (Pearson) for the chemical and biochemical variables.

|  | IAA | IAAlike | GAlike | IPAlike | Aliph-C | Pepti-C | Arom-C | Phen-C | Carb-C | DW | NO_3_^-^ | SO_4_^2-^ | Fe | K | Mg | Ca | INV | POX | EST | NR | GS | OAS | Asp | Thr | Ile | Lys | Asn | Glu | Met | Cys | Ser | Aliin |
| --- | --- | --- | --- | --- | --- | --- | --- | --- | --- | --- | --- | --- | --- | --- | --- | --- | --- | --- | --- | --- | --- | --- | --- | --- | --- | --- | --- | --- | --- | --- | --- | --- |
| AD | 0.86^**^ | 0.83^**^ | 0.91^**^ | 0.85^**^ | -0.93^**^ | -0.88^**^ | 0.94^**^ | nd | 0.95^**^ | nd | nd | nd | nd | nd | nd | nd | nd | nd | nd | nd | nd | nd | nd | nd | nd | nd | nd | nd | nd | nd | nd | nd |
| HI/HB | -0.71^**^ | -0.68^**^ | -0.75^**^ | -0.72^**^ | nd | 0.91^**^ | -0.82^**^ | 0.64^**^ | -0.80^**^ | nd | nd | nd | nd | nd | nd | nd | nd | 0.53^*^ | nd | nd | nd | 0.48^*^ | nd | 0.49^*^ | nd | nd | nd | nd | nd | nd | nd | nd |
| Ns | -0.56^*^ | -0.50^*^ | -0.77^**^ | -0.82^**^ | 0.77^**^ | 0.54^*^ | -0.65^**^ | nd | -0.65^**^ | nd | -0.58^*^ | -0.57^*^ | nd | -0.71^**^ | nd | -0.60^**^ | -0.72^**^ | -0.57^*^ | -0.57^*^ | -0.60^**^ | -0.66^**^ | -0.57^*^ | -0.58^*^ | -0.62^*^ | -0.68^**^ | -0.60^*^ | -0.70^**^ | nd | nd | --0.66^**^ | -0.71^**^ | -0.66^**^ |
| C | -0.82 | -0.78^**^ | -0.93^**^ | -0.87^**^ | 0.76^**^ | 0.75^**^ | -0.79^**^ | nd | -0.82^**^ | nd | nd | nd | nd | -0.62^**^ | nd | -0.63^**^ | -0.55^*^ | nd | -0.55^*^ | -0.53^*^ | nd | nd | -0.54^*^ | nd | -0.64^**^ | -0.55^*^ | -0.68^**^ | nd | nd | -0.56^*^ | -0.52^*^ | -.579^*^ |
| H | nd | nd | nd | 0.63^**^ | nd | -0.58^*^ | 0.60^**^ | -0.77^**^ | 0.53^*^ | -0.55^*^ | -0.55^*^ | -0.56^*^ | -0.58^*^ | nd | -0.61^**^ | -0.50^*^ | nd | nd | -0.55^*^ | -0.53^*^ | nd | -0.55^*^ | -0.50^*^ | -0.47^*^ | nd | -0.54^*^ | nd | -0.50^*^ | -0.51^*^ | -0.48^*^ | nd | -0.48^*^ |
| N | 0.95^**^ | 0.94^**^ | 0.88^**^ | 0.74^**^ | -0.66^**^ | -0.95^**^ | 0.90^**^ | nd | 0.93^**^ | nd | nd | nd | nd | nd | nd | nd | nd | nd | nd | nd | nd | nd | nd | nd | nd | nd | nd | nd | nd | nd | nd | nd |
| O | -0.62^**^ | -0.54^*^ | -0.85^**^ | -0.93^**^ | 0.60^**^ | 0.73^**^ | -0.76^**^ | 0.56^*^ | -0.75^**^ | nd | nd | nd | nd | nd | nd | nd | nd | nd | nd | nd | nd | nd | nd | nd | nd | nd | nd | nd | nd | nd | nd | nd |
| S | -0.53^*^ | nd | -0.73^**^ | -0.84^**^ | 0.84^**^ | 0.59^**^ | -0.71^**^ | nd | -0.70^**^ | nd | nd | nd | nd | nd | nd | nd | -0.50^*^ | nd | nd | nd | nd | nd | nd | nd | nd | nd | nd | nd | nd | nd | -0.51^*^ | nd |
| COOH | 0.89^**^ | 0.89^**^ | 0.81^**^ | 0.65^**^ | -0.54^*^ | -0.91^**^ | 0.84^**^ | nd | 0.87^**^ | nd | nd | nd | nd | nd | nd | nd | nd | nd | nd | nd | nd | nd | nd | nd | nd | nd | nd | nd | nd | nd | nd | nd |
| PH-OH | 0.89^**^ | 0.93^**^ | 0.71^**^ | nd | -0.70^**^ | -0.63^**^ | 0.62^**^ | nd | 0.71^**^ | 0.56^*^ | nd | nd | nd | nd | nd | 0.47^*^ | nd | nd | nd | nd | nd | nd | nd | nd | 0.52^*^ | nd | nd | nd | nd | nd | nd | nd |
| IAA | 1 | 0.99^**^ | 0.93^**^ | 0.75^**^ | -0.76^**^ | -0.89^**^ | 0.86^**^ | nd | 0.93^**^ | nd | nd | nd | nd | nd | nd | nd | nd | nd | nd | nd | nd | nd | nd | nd | nd | nd | nd | nd | nd | nd | nd | nd |
| IAAlike |  | 1 | 0.89^**^ | 0.67^**^ | -0.74^**^ | -0.85^**^ | 0.83^**^ | nd | 0.89^**^ | nd | nd | nd | nd | nd | nd | nd | nd | nd | nd | nd | nd | nd | nd | nd | nd | nd | nd | nd | nd | nd | nd | nd |
| GAlike |  |  | 1 | 0.92^**^ | -0.81^**^ | -0.93^**^ | 0.94^**^ | nd | 0.97^**^ | nd | nd | nd | nd | nd | nd | nd | nd | nd | nd | nd | nd | nd | nd | nd | nd | nd | nd | nd | nd | nd | nd | nd |
| IPAlike |  |  |  | 1 | -0.74^**^ | -0.88^**^ | 0.91^**^ | -0.59^**^ | 0.90^**^ | nd | nd | nd | nd | nd | nd | nd | nd | nd | nd | nd | nd | nd | nd | nd | nd | nd | nd | nd | nd | nd | nd | nd |
| Alph-C |  |  |  |  | 1 | 0.69^**^ | -0.81^**^ | nd | -0.82^**^ | nd | nd | nd | nd | nd | nd | nd | nd | nd | nd | nd | nd | nd | nd | nd | -0.51^*^ | nd | nd | nd | nd | nd | nd | nd |
| Pepti-C |  |  |  |  |  | 1 | -0.97^**^ | 0.55^*^ | -0.97^**^ | nd | nd | nd | nd | nd | nd | nd | nd | nd | nd | nd | nd | nd | nd | nd | nd | nd | nd | nd | nd | nd | nd | nd |
| Arom-C |  |  |  |  |  |  | 1 | -0.58^*^ | 0.98^**^ | nd | nd | nd | nd | nd | nd | nd | nd | nd | nd | nd | nd | nd | nd | nd | nd | nd | nd | nd | nd | nd | nd | nd |
| Phen-Cl |  |  |  |  |  |  |  | 1 | nd | nd | nd | nd | nd | nd | nd | nd | nd | nd | nd | nd | nd | nd | nd | nd | nd | nd | nd | nd | nd | nd | nd | nd |
| Carb-C |  |  |  |  |  |  |  |  | 1 | nd | nd | nd | nd | nd | nd | nd | nd | nd | nd | nd | nd | nd | nd | nd | nd | nd | nd | nd | nd | nd | nd | nd |
| DW |  |  |  |  |  |  |  |  |  | 1 | 0.73^**^ | 0.75^**^ | 0.77^**^ | 0.81^**^ | 0.78^**^ | 0.88^**^ | 0.75^**^ | 0.61^**^ | 0.93^**^ | 0.88^**^ | 0.68^**^ | 0.80^**^ | 0.80^**^ | 0.77^**^ | 0.75^**^ | 0.80^**^ | 0.75^**^ | 0.72^**^ | 0.88^**^ | 0.76^**^ | 0.78^**^ | 0.76^**^ |
| NO_3_ |  |  |  |  |  |  |  |  |  |  | 1 | 0.99^**^ | 0.93^**^ | 0.95^**^ | 0.93^**^ | 0.88^**^ | 0.92^**^ | 0.96^**^ | 0.90^**^ | 0.95^**^ | 0.96^**^ | 0.97^**^ | 0.90^**^ | 0.86^**^ | 0.88^**^ | 0.96^**^ | 0.89^**^ | 0.88^**^ | 0.87^**^ | 0.98^**^ | 0.88^**^ | 0.94^**^ |
| SO_4_^2^ |  |  |  |  |  |  |  |  |  |  |  | 1 | 0.92^**^ | 0.96^**^ | 0.94^**^ | 0.89^**^ | 0.91^**^ | 0.95^**^ | 0.91^**^ | 0.96^**^ | 0.96^**^ | 0.97^**^ | 0.90^**^ | 0.88^**^ | 0.90^**^ | 0.96^**^ | 0.89^**^ | 0.86^**^ | 0.87^**^ | 0.97^**^ | 0.88^**^ | 0.94^**^ |
| Fe |  |  |  |  |  |  |  |  |  |  |  |  | 1 | 0.89^**^ | 0.92^**^ | 0.87^**^ | 0.87^**^ | 0.87^**^ | 0.89^**^ | 0.91^**^ | 0.88^**^ | 0.91^**^ | 0.87^**^ | 0.82^**^ | 0.80^**^ | 0.91^**^ | 0.86^**^ | 0.89^**^ | 0.87^**^ | 0.92^**^ | 0.84^**^ | 0.85^**^ |
| K |  |  |  |  |  |  |  |  |  |  |  |  |  | 1 | 0.91^**^ | 0.95^**^ | 0.93^**^ | 0.89^**^ | 0.95^**^ | 0.98^**^ | 0.93^**^ | 0.95^**^ | 0.93^**^ | 0.86^**^ | 0.93^**^ | 0.97^**^ | 0.93^**^ | 0.82^**^ | 0.87^**^ | 0.97^**^ | 0.92^**^ | 0.96^**^ |
| Mg |  |  |  |  |  |  |  |  |  |  |  |  |  |  | 1 | 0.92^**^ | 0.83^**^ | 0.88^**^ | 0.91^**^ | 0.94^**^ | 0.87^**^ | 0.89^**^ | 0.88^**^ | 0.74^**^ | 0.78^**^ | 0.96^**^ | 0.84^**^ | 0.82^**^ | 0.90^**^ | 0.95^**^ | 0.84^**^ | 0.93^**^ |
| Ca |  |  |  |  |  |  |  |  |  |  |  |  |  |  |  | 1 | 0.84^**^ | 0.77^**^ | 0.97^**^ | 0.96^**^ | 0.80^**^ | 0.87^**^ | 0.92^**^ | 0.76^**^ | 0.89^**^ | 0.96^**^ | 0.90^**^ | 0.76^**^ | 0.86^**^ | 0.93^**^ | 0.83^**^ | 0.92^**^ |
| INV |  |  |  |  |  |  |  |  |  |  |  |  |  |  |  |  | 1 | 0.91^**^ | 0.88^**^ | 0.92^**^ | 0.95^**^ | 0.94^**^ | 0.88^**^ | 0.87^**^ | 0.84^**^ | 0.90^**^ | 0.88^**^ | 0.85^**^ | 0.87^**^ | 0.92^**^ | 0.94^**^ | 0.91^**^ |
| POX |  |  |  |  |  |  |  |  |  |  |  |  |  |  |  |  |  | 1 | 0.80^**^ | 0.88^**^ | 0.98^**^ | 0.94^**^ | 0.85^**^ | 0.85^**^ | 0.80^**^ | 0.89^**^ | 0.78^**^ | 0.85^**^ | 0.84^**^ | 0.92^**^ | 0.88^**^ | 0.90^**^ |
| EST |  |  |  |  |  |  |  |  |  |  |  |  |  |  |  |  |  |  | 1 | 0.98^**^ | 0.85^**^ | 0.92^**^ | 0.91^**^ | 0.85^**^ | 0.89^**^ | 0.95^**^ | 0.90^**^ | 0.82^**^ | 0.92^**^ | 0.93^**^ | 0.88^**^ | 0.92^**^ |
| NR |  |  |  |  |  |  |  |  |  |  |  |  |  |  |  |  |  |  |  | 1 | 0.91^**^ | 0.95^**^ | 0.93^**^ | 0.87^**^ | 0.90^**^ | 0.98^**^ | 0.90^**^ | 0.85^**^ | 0.93^**^ | 0.97^**^ | 0.91^**^ | 0.96^**^ |
| GS |  |  |  |  |  |  |  |  |  |  |  |  |  |  |  |  |  |  |  |  | 1 | 0.95^**^ | 0.84^**^ | 0.86^**^ | 0.82^**^ | 0.91^**^ | 0.84^**^ | .858^**^ | 0.87^**^ | 0.94^**^ | 0.93^**^ | 0.92^**^ |
| OAS-s |  |  |  |  |  |  |  |  |  |  |  |  |  |  |  |  |  |  |  |  |  | 1 | 0.92^**^ | 0.94^**^ | 0.90^**^ | 0.94^**^ | 0.85^**^ | 0.89^**^ | 0.89^**^ | 0.94^**^ | 0.89^**^ | 0.91^**^ |
| Asp |  |  |  |  |  |  |  |  |  |  |  |  |  |  |  |  |  |  |  |  |  |  | 1 | 0.84^**^ | 0.89^**^ | 0.93^**^ | 0.83^**^ | 0.80^**^ | 0.82^**^ | 0.91^**^ | 0.83^**^ | 0.91^**^ |
| Thr |  |  |  |  |  |  |  |  |  |  |  |  |  |  |  |  |  |  |  |  |  |  |  | 1 | 0.85^**^ | 0.82^**^ | 0.74^**^ | 0.86^**^ | 0.82^**^ | 0.82^**^ | 0.81^**^ | 0.77^**^ |
| Ile |  |  |  |  |  |  |  |  |  |  |  |  |  |  |  |  |  |  |  |  |  |  |  |  | 1 | 0.89^**^ | 0.90^**^ | 0.72^**^ | 0.72^**^ | 0.89^**^ | 0.77^**^ | 0.87^**^ |
| Lys |  |  |  |  |  |  |  |  |  |  |  |  |  |  |  |  |  |  |  |  |  |  |  |  |  | 1 | 0.91^**^ | 0.86^**^ | 0.89^**^ | 0.98^**^ | 0.87^**^ | 0.96^**^ |
| Asn |  |  |  |  |  |  |  |  |  |  |  |  |  |  |  |  |  |  |  |  |  |  |  |  |  |  | 1 | 0.75^**^ | 0.76^**^ | 0.93^**^ | 0.81^**^ | 0.89^**^ |
| Glu |  |  |  |  |  |  |  |  |  |  |  |  |  |  |  |  |  |  |  |  |  |  |  |  |  |  |  | 1 | 0.88^**^ | 0.85^**^ | 0.79^**^ | 0.75^**^ |
| Met |  |  |  |  |  |  |  |  |  |  |  |  |  |  |  |  |  |  |  |  |  |  |  |  |  |  |  |  | 1 | 0.87^**^ | 0.91^**^ | 0.87^**^ |
| Cys |  |  |  |  |  |  |  |  |  |  |  |  |  |  |  |  |  |  |  |  |  |  |  |  |  |  |  |  |  | 1 | 0.90^**^ | 0.96^**^ |
| Ser |  |  |  |  |  |  |  |  |  |  |  |  |  |  |  |  |  |  |  |  |  |  |  |  |  |  |  |  |  |  | 1 | 0.89^**^ |

AD, aromaticity dregree; Alph-C, aliphatic-C; Arom-C, aromatic-C; Asn, asparagine; Asp, aspartate; C, carbon; Ca, calcium; Carb-C, carboxyl-C; COOH, carboxylic acidity; Cys, cysteine; DW, root dry weight; EST, esterase activity; Fe, iron; GA-like, gibberellin-like activity; Glu, glutamate; GS, glutamime synthetase activity; H, hydrogen; HI/HB, hydrophobic index; IAA, indoleacetic acid content; IAA-like, auxin-like activity; Ile, isoleucine; INV, invertase activity; IPA-like, cytokinin-like activity; K, potassium; Lys, lysine; Met, methionine; Mg, magnesium; N, nitrogen; NO_3_^-^, nitrate; NR, nitrate reductase activity; Ns, nominal size of HS; O, oxygen; OAS-s, O-acetylserine sulphydrilase activity; Pepti-C, peptidic-C, PH-OH, Phenolic acidity; Phen-C, Phenoli-C; POX, peroxidase activity; S, sulfur; Ser, serine; SO_4_^2-^, sulfate; Thr, threonine.

^*^ Significance at *p* ≤0.05; ^**^ Significance at *p* ≤0.01; Nd, not significant

**Table S2** Parameters of the regression curves (first and second traits) between concentration of humic substances (total humic extract, THE, high molecular size, HMS and low molecular size, LMS) and root biomass and macro and micro nutrients content in HS-treated garlic plantlets.

|  | **First trait** | | | | | | **Second trait** | | | | | |
| --- | --- | --- | --- | --- | --- | --- | --- | --- | --- | --- | --- | --- |
| **Humic substance** | **R2** | **SE** | **DF** | ***P*** | **b** | **R2** | | **SE** | **DF** | **P** | **b** |  |
|  | Root biomass | | | | | | | | | | | |
| THE1 | 0.75 | 0.80 | 13 | 0.000 | 3.60 | 0.897 | | 0.75 | 7 | 0.000 | -1.14 |  |
| HMS1 | 0.783 | 2.19 | 10 | 0.000 | 20.22 | 0.923 | | 1.12 | 10 | 0.000 | -2.82 |  |
| LMS1 | 0.917 | 1.04 | 7 | 0.000 | 29.59 | 0.798 | | 1.30 | 12 | 0.000 | -1.35 |  |
| THE2 | 0.670 | 3.53 | 13 | 0.000 | 13.10 | 0.768 | | 1.94 | 7 | 0.002 | -1.83 |  |
| HMS2 | 0.928 | 2.89 | 10 | 0.000 | 50.31 | 0.902 | | 2.18 | 10 | 0.000 | -3.47 |  |
| LMS2 | 0.874 | 3.70 | 7 | 0.000 | 83.63 | 0.565 | | 3.50 | 13 | 0.001 | -2.14 |  |
|  | Nitrate | | | | | | | | | | | |
| THE1 | 0.887 | 0.62 | 13 | 0.000 | 4.52 | 0.943 | | 1.56 | 7 | 0.000 | -3.27 |  |
| HMS1 | 0.953 | 1.43 | 10 | 0.000 | 31.21 | 0.841 | | 3.12 | 10 | 0.000 | -3.76 |  |
| LMS1 | 0.842 | 1.33 | 7 | 0.000 | 26.29 | 0.812 | | 2.18 | 12 | 0.000 | -2.36 |  |
| THE2 | 0.685 | 1.58 | 13 | 0.000 | 6.06 | 0.938 | | 0.94 | 7 | 0.000 | -1.89 |  |
| HMS2 | 0.985 | 1.18 | 10 | 0.000 | 47.01 | 0.900 | | 2.97 | 10 | 0.000 | -4.46 |  |
| LMS2 | 0.951 | 1.26 | 7 | 0.000 | 47.71 | 0.727 | | 3.52 | 13 | 0.000 | -3.09 |  |
|  | Sulfate | | | | | | | | | | | |
| THE1 | 0.854 | 0.77 | 13 | 0.000 | 4.90 | 0.876 | | 1.03 | 7 | 0.000 | -1.41 |  |
| HMS1 | 0.726 | 6.06 | 10 | 0.000 | 47.85 | 0.845 | | 2.88 | 10 | 0.000 | -3.52 |  |
| LMS1 | 0.931 | 0.94 | 7 | 0.000 | 29.65 | 0.760 | | 2.02 | 12 | 0.000 | -1.89 |  |
| THE2 | 0.800 | 0.83 | 13 | 0.000 | 4.35 | 0.816 | | 0.71 | 7 | 0.001 | -0.78 |  |
| HMS2 | 0.941 | 5.08 | 10 | 0.000 | 98.56 | 0.624 | | 10.22 | 10 | 0.002 | -6.88 |  |
| LMS2 | 0.961 | 2.04 | 7 | 0.000 | 87.63 | 0.696 | | 4.16 | 13 | 0.000 | -3.38 |  |
|  | Iron | | | | | | | | | | | |
| THE1 | 0.819 | 0.03 | 13 | 0.000 | 0.17 | 0.711 | | 0.06 | 7 | 0.004 | -0.04 |  |
| HMS1 | 0.754 | 0.07 | 10 | 0.000 | 0.57 | 0.835 | | 0.07 | 10 | 0.000 | -0.08 |  |
| LMS1 | 0.910 | 0.02 | 7 | 0.000 | 0.69 | 0.571 | | 0.12 | 12 | 0.002 | -0.07 |  |
| THE2 | 0.754 | 0.04 | 13 | 0.000 | 0.16 | 0.952 | | 0.03 | 7 | 0.000 | -0.06 |  |
| HMS2 | 0.852 | 0.07 | 10 | 0.000 | 0.80 | 0.760 | | 0.08 | 10 | 0.000 | -0.08 |  |
| LMS2 | 0.943 | 0.02 | 7 | 0.000 | 0.98 | 0.762 | | 0.06 | 13 | 0.000 | -0.06 |  |
|  | Potassium | | | | | | | | | | | |
| THE1 | 0.589 | 0.89 | 13 | 0.001 | 2.83 | 0.919 | | 1.36 | 7 | 0.000 | -2.38 |  |
| HMS1 | 0.855 | 3.48 | 10 | 0.000 | 40.97 | 0.840 | | 4.04 | 10 | 0.000 | -4.84 |  |
| LMS1 | 0.933 | 1.55 | 7 | 0.000 | 49.77 | 0.744 | | 3.13 | 12 | 0.000 | -2.81 |  |
| THE2 | 0.856 | 0.88 | 13 | 0.000 | 5.63 | 0.979 | | 0.77 | 7 | 0.000 | -2.74 |  |
| HMS2 | 0.977 | 2.68 | 10 | 0.000 | 85.76 | 0.651 | | 11.15 | 10 | 0.002 | -7.96 |  |
| LMS2 | 0.955 | 2.76 | 7 | 0.000 | 109.72 | 0.653 | | 7.11 | 13 | 0.000 | -5.25 |  |
|  | Magnesium | | | | | | | | | | | |
| THE1 | 0.791 | 0.62 | 13 | 0.000 | 3.15 | 0.629 | | 0.97 | 7 | 0.011 | -0.65 |  |
| HMS1 | 0.865 | 0.88 | 10 | 0.000 | 10.84 | 0.764 | | 1.25 | 10 | 0.000 | -1.17 |  |
| LMS1 | 0.765 | 0.74 | 7 | 0.002 | 11.49 | 0.681 | | 1.43 | 12 | 0.000 | -1.09 |  |
| THE2 | 0.624 | 0.44 | 13 | 0.000 | 1.49 | 0.915 | | 0.51 | 7 | 0.000 | -0.87 |  |
| HMS2 | 0.958 | 1.80 | 10 | 0.000 | 41.75 | 0.844 | | 2.65 | 10 | 0.000 | -3.22 |  |
| LMS2 | 0.935 | 0.91 | 7 | 0.000 | 29.61 | 0.808 | | 1.73 | 13 | 0.000 | -1.90 |  |
|  | Calcium | | | | | | | | | | | |
| THE1 | 0.969 | 0.87 | 13 | 0.000 | 11.94 | 0.952 | | 1.05 | 7 | 0.000 | -2.43 |  |
| HMS1 | 0.985 | 0.86 | 10 | 0.000 | 33.68 | 0.741 | | 1.86 | 10 | 0.000 | -1.64 |  |
| LMS1 | 0.787 | 3.31 | 7 | 0.001 | 54.61 | 0.810 | | 1.94 | 10 | 0.000 | -2.11 |  |
| THE2 | 0.959 | 1.09 | 13 | 0.000 | 13.69 | 0.917 | | 1.63 | 7 | 0.000 | -2.81 |  |
| HMS2 | 0.918 | 3.86 | 10 | 0.000 | 62.45 | 0.481 | | 9.40 | 10 | 0.012 | -4.74 |  |
| LMS2 | 0.990 | 1.13 | 7 | 0.000 | 96.24 | 0.445 | | 7.27 | 13 | 0.007 | -3.49 |  |

R2 = R squared; SE = Standard Error; DF = Degrees of Freedom; b = coefficient of regression

**Table S3** Parameters of the regression curves (first and second traits) between concentration of humic substances (total humic extract, THE, high molecular size, HMS and low molecular size, LMS) and enzyme activities in roots of HS-treated garlic plantlets.

|  | **First trait** | | | | | **Second trait** | | | | |
| --- | --- | --- | --- | --- | --- | --- | --- | --- | --- | --- |
| **Humic substances** | **R2** | **SE** | **DF** | **P** | **b** | **R2** | **SE** | **DF** | **P** | **b** |
|  | Nitrate reductase | | | | | | | | | |
| THE1 | 0.658 | 1.16 | 13 | 0.000 | 4.22 | 0.974 | 0.40 | 7 | 0.000 | -1.26 |
| HMS1 | 0.968 | 1.26 | 10 | 0.000 | 33.87 | 0.954 | 0.99 | 10 | 0.000 | -2.36 |
| LMS1 | 0.924 | 1.29 | 7 | 0.000 | 38.66 | 0.644 | 1.91 | 12 | 0.001 | -1.35 |
| THE2 | 0.867 | 1.31 | 13 | 0.000 | 8.71 | 0.862 | 1.87 | 7 | 0.000 | -2.43 |
| HMS2 | 0.821 | 6.19 | 10 | 0.000 | 64.27 | 0.695 | 7.63 | 10 | 0.001 | -6.03 |
| LMS2 | 0.967 | 1.83 | 7 | 0.000 | 85.23 | 0.700 | 3.28 | 13 | 0.000 | -2.70 |
|  | Glutamate synthetase | | | | | | | | |  |
| THE1 | 0.989 | 0.41 | 13 | 0.000 | 10.14 | 0.853 | 1.57 | 7 | 0.000 | -1.96 |
| HMS1 | 0.993 | 0.87 | 10 | 0.000 | 51.19 | 0.812 | 3.35 | 10 | 0.000 | -3.64 |
| LMS1 | 0.979 | 1.16 | 7 | 0.000 | 69.15 | 0.792 | 2.77 | 12 | 0.000 | -2.85 |
| THE2 | 0.969 | 0.90 | 13 | 0.000 | 13.10 | 0.902 | 1.15 | 7 | 0.000 | -1.81 |
| HMS2 | 0.981 | 1.89 | 10 | 0.000 | 66.85 | 0.857 | 4.51 | 10 | 0.000 | -5.77 |
| LMS2 | 0.905 | 2.77 | 7 | 0.000 | 73.43 | 0.668 | 4.08 | 13 | 0.000 | -3.11 |
|  | O-acetylserine sulphydrilase | | | | | | | | | |
| THE1 | 0.883 | 1.96 | 13 | 0.000 | 14.04 | 0.933 | 1.39 | 7 | 0.000 | -2.68 |
| HMS1 | 0.976 | 3.21 | 10 | 0.000 | 99.91 | 0.715 | 11.09 | 10 | 0.001 | -9.18 |
| LMS1 | 0.946 | 2.61 | 7 | 0.000 | 94.05 | 0.771 | 4.88 | 12 | 0.000 | -4.72 |
| THE2 | 0.790 | 4.71 | 13 | 0.000 | 23.86 | 0.941 | 3.17 | 7 | 0.000 | -6.61 |
| HMS2 | 0.963 | 5.72 | 10 | 0.000 | 142.02 | 0.771 | 9.02 | 10 | 0.000 | -8.66 |
| LMS2 | 0.887 | 6.95 | 7 | 0.000 | 167.20 | 0.846 | 7.98 | 13 | 0.000 | -10.05 |
|  | Invertase | | | | | | | | | |
| THE1 | 0.718 | 0.09 | 13 | 0.000 | 0.37 | 0.925 | 0.06 | 7 | 0.000 | -0.11 |
| HMS1 | 0.981 | 0.05 | 10 | 0.000 | 1.66 | 0.842 | 0.14 | 10 | 0.000 | -0.17 |
| LMS1 | 0.909 | 0.10 | 7 | 0.000 | 2.68 | 0.971 | 0.09 | 12 | 0.000 | -0.27 |
| THE2 | 0.594 | 0.14 | 13 | 0.001 | 0.45 | 0.916 | 0.07 | 7 | 0.000 | -0.12 |
| HMS2 | 0.981 | 0.06 | 10 | 0.000 | 2.13 | 0.697 | 0.19 | 10 | 0.001 | -0.15 |
| LMS2 | 0.968 | 0.07 | 7 | 0.000 | 3.12 | 0.873 | 0.14 | 13 | 0.000 | -0.19 |
|  | Peroxidase | | | | | | | | | |
| THE1 | 0.942 | 0.03 | 13 | 0.000 | 0.36 | 0.978 | 0.02 | 7 | 0.000 | -0.08 |
| HMS1 | 0.984 | 0.08 | 10 | 0.000 | 3.08 | 0.710 | 0.27 | 10 | 0.001 | -0.22 |
| LMS1 | 0.962 | 0.06 | 7 | 0.000 | 2.90 | 0.834 | 0.09 | 12 | 0.000 | -0.11 |
| THE2 | 0.863 | 0.08 | 13 | 0.000 | 0.53 | 0.863 | 0.09 | 7 | 0.000 | -0.12 |
| HMS2 | 0.978 | 0.13 | 10 | 0.000 | 4.42 | 0.727 | 0.33 | 10 | 0.000 | -0.28 |
| LMS2 | 0.657 | 0.44 | 7 | 0.050 | 2.81 | 0.936 | 0.07 | 13 | 0.000 | -0.15 |
|  | Esterase | | | | | | | | | |
| THE1 | 0.922 | 0.03 | 13 | 0.000 | 0.27 | 0.818 | 0.03 | 7 | 0.001 | -0.04 |
| HMS1 | 0.902 | 0.11 | 10 | 0.000 | 1.58 | 0.928 | 0.07 | 10 | 0.000 | -0.12 |
| LMS1 | 0.984 | 0.03 | 7 | 0.000 | 1.83 | 0.468 | 0.14 | 12 | 0.007 | -0.07 |
| THE2 | 0.964 | 0.04 | 13 | 0.000 | 0.53 | 0.839 | 0.10 | 7 | 0.001 | -0.12 |
| HMS2 | 0.974 | 0.10 | 10 | 0.000 | 3.09 | 0.870 | 0.17 | 10 | 0.000 | -0.23 |
| LMS2 | 0.942 | 0.13 | 7 | 0.000 | 4.45 | 0.851 | 0.15 | 13 | 0.000 | -0.19 |

R2 = R squared; SE = Standard Error; DF = Degrees of Freedom; b = coefficient of regression

**Table S4** Parameters of the regression curves (first and second traits) between concentration of humic substances (total humic extract, THE, high molecular size, HMS and low molecular size, LMS) and amino acid content in roots of HS-treated garlic plants.

|  | **First trait** | | | | | **Second trait** | | | | | |
| --- | --- | --- | --- | --- | --- | --- | --- | --- | --- | --- | --- |
| **Humic substance** | **R2** | **SE** | **DF** | **P** | **b** | **R2** | **SE** | **DF** | **P** | **b** |  |
|  | Aspartate | | | | | | | | | | |
| THE1 | 0.531 | 0.24 | 13 | 0.031 | 0.42 | 0.927 | 0.15 | 7 | 0.000 | -0.29 |  |
| HMS1 | 0.855 | 0.07 | 10 | 0.000 | 0.81 | 0.991 | 0.05 | 10 | 0.000 | -0.30 |  |
| LMS1 | 0.759 | 0.05 | 7 | 0.002 | 0.78 | 0.854 | 0.19 | 12 | 0.000 | -0.241 |  |
| THE2 | 0.455 | 0.27 | 13 | 0.018 | 0.53 | 0.979 | 0.49 | 7 | 0.000 | -0.17 |  |
| HMS2 | 0.590 | 0.30 | 10 | 0.004 | 1.77 | 0.744 | 0.29 | 10 | 0.000 | -0.26 |  |
| LMS2 | 0.747 | 0.12 | 7 | 0.003 | 1.85 | 0.721 | 0.31 | 13 | 0.000 | -0.27 |  |
|  | Threonine | | | | | | | | | | |
| THE1 | 0.308 | 0.16 | 13 | 0.032 | 0.29 | 0.906 | 0.11 | 7 | 0.000 | -0.17 |  |
| HMS1 | 0.716 | 0.20 | 10 | 0.001 | 1.52 | 0.858 | 0.34 | 10 | 0.000 | -0.44 |  |
| LMS1 | 0.610 | 0.08 | 7 | 0.013 | 0.95 | 0.715 | 0.33 | 12 | 0.000 | -0.281 |  |
| THE2 | 0.450 | 0.32 | 13 | 0.006 | 0.75 | 0.952 | 0.14 | 7 | 0.000 | -0.33 |  |
| HMS2 | 0.689 | 0.28 | 10 | 0.001 | 2.03 | 0.849 | 0.29 | 10 | 0.000 | -0.36 |  |
| LMS2 | 0.931 | 0.06 | 7 | 0.000 | 2.09 | 0.791 | 0.35 | 13 | 0.000 | -0.37 |  |
|  | Isoleucine | | | | | | | | | | |
| THE1 | 0.620 | 0.07 | 13 | 0.000 | 0.24 | 0.905 | 0.08 | 7 | 0.000 | -0.14 |  |
| HMS1 | 0.765 | 0.20 | 10 | 0.000 | 1.78 | 0.829 | 0.15 | 10 | 0.000 | -0.17 |  |
| LMS1 | 0.914 | 0.03 | 7 | 0.000 | 0.99 | 0.833 | 0.15 | 12 | 0.000 | -0.18 |  |
| THE2 | 0.425 | 0.20 | 13 | 0.008 | 0.44 | 0.851 | 0.17 | 7 | 0.000 | -0.21 |  |
| HMS2 | 0.499 | 0.32 | 10 | 0.010 | 1.56 | 0.862 | 0.20 | 10 | 0.000 | -0.25 |  |
| LMS2 | 0.920 | 0.11 | 7 | 0.000 | 3.30 | 0.715 | 0.33 | 13 | 0.000 | -0.28 |  |
|  | Lysine | | | | | | | | | | |
| THE1 | 0.358 | 0.19 | 13 | 0.019 | 0.38 | 0.923 | 0.07 | 7 | 0.000 | -0.12 |  |
| HMS1 | 0.779 | 0.15 | 10 | 0.000 | 1.39 | 0.814 | 0.27 | 10 | 0.000 | -0.29 |  |
| LMS1 | 0.654 | 0.10 | 7 | 0.008 | 1.22 | 0.763 | 0.16 | 12 | 0.000 | -0.15 |  |
| THE2 | 0.336 | 0.20 | 13 | 0.023 | 0.37 | 0.786 | 0.17 | 7 | 0.001 | -0.16 |  |
| HMS2 | 0.939 | 0.14 | 10 | 0.000 | 2.72 | 0.726 | 0.41 | 10 | 0.000 | -0.35 |  |
| LMS2 | 0.917 | 0.10 | 7 | 0.000 | 3.00 | 0.629 | 0.32 | 13 | 0.000 | -0.22 |  |
|  | Asparagine | | | | | | | | | | |
| THE1 | 0.350 | 0.44 | 13 | 0.020 | 0.85 | 0.907 | 0.26 | 7 | 0.000 | -0.43 |  |
| HMS1 | 0.926 | 0.11 | 10 | 0.000 | 1.90 | 0.873 | 0.26 | 10 | 0.000 | -0.36 |  |
| LMS1 | 0.869 | 0.09 | 7 | 0.000 | 2.12 | 0.842 | 0.33 | 12 | 0.000 | -0.41 |  |
| THE2 | 0.332 | 0.35 | 13 | 0.025 | 0.65 | 0.924 | 0.16 | 7 | 0.000 | -0.30 |  |
| HMS2 | 0.883 | 0.22 | 10 | 0.000 | 2.89 | 0.970 | 0.17 | 10 | 0.000 | -0.51 |  |
| LMS2 | 0.915 | 0.15 | 7 | 0.000 | 4.41 | 0.811 | 0.38 | 13 | 0.000 | -0.42 |  |
|  | Glutamate | | | | | | | | | | |
| THE1 | 0.208 | 0.57 | 13 | 0.088 | 0.77 | 0.796 | 0.40 | 7 | 0.001 | -0.41 |  |
| HMS1 | 0.391 | 0.58 | 10 | 0.030 | 2.25 | 0.847 | 0.28 | 10 | 0.000 | -0.34 |  |
| LMS1 | 0.180 | 0.29 | 7 | 0.255 | 1.18 | 0.839 | 0.32 | 12 | 0.000 | -0.39 |  |
| THE2 | 0.341 | 0.45 | 13 | 0.022 | 0.85 | 0.928 | 0.19 | 7 | 0.000 | -0.36 |  |
| HMS2 | 0.861 | 0.21 | 10 | 0.000 | 2.50 | 0.734 | 0.58 | 10 | 0.000 | -0.51 |  |
| LMS2 | 0.371 | 0.29 | 7 | 0.082 | 1.91 | 0.583 | 0.47 | 13 | 0.001 | -0.30 |  |
|  | Methionine | | | | | | | | | | |
| THE1 | 0.634 | 0.46 | 13 | 0.000 | 1.57 | 0.979 | 0.29 | 7 | 0.000 | -1.03 |  |
| HMS1 | 0.895 | 0.41 | 10 | 0.000 | 5.82 | 0.904 | 0.47 | 10 | 0.000 | -0.76 |  |
| LMS1 | 0.762 | 0.59 | 7 | 0.002 | 9.04 | 0.837 | 0.73 | 12 | 0.000 | -0.87 |  |
| THE2 | 0.865 | 0.39 | 13 | 0.000 | 2.56 | 0.975 | 0.23 | 7 | 0.000 | -0.74 |  |
| HMS2 | 0.863 | 0.92 | 10 | 0.000 | 11.14 | 0.907 | 0.79 | 10 | 0.000 | -1.29 |  |
| LMS2 | 0.883 | 0.51 | 7 | 0.000 | 12.06 | 0.654 | 1.06 | 13 | 0.000 | -0.78 |  |
|  | Cysteine | | | | | | | | | | |
| THE1 | 0.685 | 0.44 | 13 | 0.000 | 1.72 | 0.936 | 0.42 | 7 | 0.000 | -0.84 |  |
| HMS1 | 0.878 | 0.45 | 10 | 0.000 | 5.84 | 0.886 | 0.61 | 10 | 0.000 | -0.89 |  |
| LMS1 | 0.943 | 0.23 | 7 | 0.000 | 7.93 | 0.717 | 0.82 | 12 | 0.000 | -0.68 |  |
| THE2 | 0.562 | 0.58 | 13 | 0.001 | 1.71 | 0.933 | 0.29 | 7 | 0.000 | -0.56 |  |
| HMS2 | 0.895 | 0.74 | 10 | 0.000 | 10.52 | 0.747 | 1.45 | 10 | 0.000 | -1.30 |  |
| LMS2 | 0.960 | 0.31 | 7 | 0.000 | 12.93 | 0.540 | 0.92 | 13 | 0.002 | -0.54 |  |
|  | Serine | | | | | | | | | | |
| THE1 | 0.642 | 1.21 | 13 | 0.000 | 4.21 | 0.918 | 0.87 | 7 | 0.000 | -1.50 |  |
| HMS1 | 0.975 | 0.43 | 10 | 0.000 | 13.28 | 0.781 | 2.27 | 10 | 0.000 | -2.25 |  |
| LMS1 | 0.937 | 0.74 | 7 | 0.000 | 24.48 | 0.865 | 1.65 | 12 | 0.000 | -2.19 |  |
| THE2 | 0.844 | 0.96 | 13 | 0.000 | 5.84 | 0.946 | 0.62 | 7 | 0.000 | -1.34 |  |
| HMS2 | 0.940 | 1.01 | 10 | 0.000 | 19.32 | 0.743 | 1.46 | 10 | 0.000 | -1.30 |  |
| LMS2 | 0.928 | 0.83 | 7 | 0.000 | 25.61 | 0.902 | 1.28 | 13 | 0.000 | -2.10 |  |
|  | Aliin | | | | | | | | | | |
| THE1 | 0.367 | 0.87 | 13 | 0.049 | 1.37 | 0.911 | 0.50 | 7 | 0.000 | -0.83 |  |
| HMS1 | 0.843 | 0.36 | 10 | 0.000 | 4.14 | 0.872 | 0.63 | 10 | 0.000 | -0.86 |  |
| LMS1 | 0.753 | 0.29 | 7 | 0.002 | 4.35 | 0.602 | 1.01 | 12 | 0.001 | -0.65 |  |
| THE2 | 0.233 | 0.61 | 13 | 0.068 | 0.87 | 0.916 | 0.40 | 7 | 0.000 | -0.69 |  |
| HMS2 | 0.954 | 0.39 | 10 | 0.000 | 8.65 | 0.916 | 0.64 | 10 | 0.000 | -1.10 |  |
| LMS2 | 0.960 | 0.21 | 7 | 0.000 | 8.76 | 0.894 | 0.50 | 13 | 0.000 | -0.78 |  |

R2 = R squared; SE = Standard Error; DF = Degrees of Freedom; b = coefficient of regression

**Table S5** Loadings values of chemical and biochemical variables on the axes identified by principal components analysis.

| **Variable** | **PC1** | **PC2** | **PC3** |
| --- | --- | --- | --- |
| **OAS-s** | 0.995 | -0.044 | 0.055 |
| **Cys** | 0.994 | -0.004 | 0.015 |
| **Lys** | 0.992 | 0.049 | 0.042 |
| **Mg** | 0.989 | -0.041 | 0.080 |
| **Sulfate** | 0.981 | -0.178 | 0.038 |
| **EST** | 0.979 | 0.162 | 0.085 |
| **GS** | 0.973 | -0.218 | 0.051 |
| **Nitrate** | 0.972 | -0.220 | 0.047 |
| **INV** | 0.970 | -0.092 | 0.002 |
| **Alliin** | 0.963 | -0.009 | 0.044 |
| **Ca** | 0.960 | 0.203 | 0.090 |
| **K** | 0.959 | 0.217 | -0.107 |
| **Met** | 0.950 | -0.165 | -0.029 |
| **Ser** | 0.946 | 0.028 | -0.120 |
| **POX** | 0.946 | -0.290 | 0.040 |
| **NR** | 0.939 | 0.315 | 0.122 |
| **Glu** | 0.919 | -0.207 | 0.105 |
| **Fe** | 0.914 | -0.127 | 0.065 |
| **Asp** | 0.903 | 0.274 | 0.128 |
| **Thr** | 0.901 | -0.122 | 0.111 |
| **Asn** | 0.897 | 0.313 | 0.148 |
| **Ile** | 0.844 | 0.416 | 0.209 |
| **DW** | 0.813 | 0.423 | 0.142 |
| **Carboxyl-C** | -0.021 | 0.999 | -0.019 |
| **N** | -0.029 | 0.998 | 0.019 |
| **IAA-like** | -0.021 | 0.998 | 0.044 |
| **IAA** | -0.020 | 0.998 | 0.047 |
| **Phenolic-OH (acidity)** | 0.026 | 0.975 | 0.213 |
| **GA-like** | 0.206 | 0.966 | -0.144 |
| **Aliphatic-C** | 0.046 | -0.949 | 0.122 |
| **COOH (acidity)** | -0.132 | 0.915 | -0.265 |
| **Aromatic-C** | -0.079 | 0.907 | -0.292 |
| **C** | -0.338 | -0.874 | 0.294 |
| **Peptidic-C** | 0.162 | -0.813 | 0.544 |
| **IPA-like** | -0.167 | 0.783 | -0.595 |
| **Phenolic-C** | 0.238 | -0.017 | 0.950 |
| **H** | -0.416 | 0.115 | -0.889 |
| **S** | -0.156 | -0.402 | 0.767 |
| **O** | -0.174 | -0.675 | 0.687 |

Asn, asparagine; Asp, aspartate; C, carbon; Ca, calcium; Cys, cysteine; EST, esterase activity; Fe, iron; GA-like, gibberellin-like activity; Glu, glutamate; GS, glutamime synthetase activity; H, hydrogen; IAA, indoleacetic acid content; IAA-like, auxin-like activity; INV, invertase activity; IPA-like, cytokinin-like activity; Ile, isoleucine; K, potassium; Lys, lysine; Met, methionine; Mg, magnesium; N, nitrogen; NO_3_^-^, nitrate; NR, nitrate reductase activity; O, oxygen; OAS-s, O-acetylserine sulphydrilase activity; POX, peroxidase activity; DW, root dry weight; S, sulfur ; Ser, serine; SO_4_^2-^, sulfate; Thr, threonine.
